# Altered Erythrocyte Function via TLR9-ox-mtDNA Binding Links Mitochondrial Oxidative Damage to Systemic Inflammation in Multiple Sclerosis

**DOI:** 10.1016/j.neurot.2026.e00863

**Published:** 2026-03-12

**Authors:** Lingfei Yang, Qianmeng Hao, Yufei Chen, Yudi Xu, Ziyi Chen, Qingsheng Li, Yaobing Yao, Zhe Gong, Huimin Liu, Hongzhuo Qin, Yanfei Li, Yanjie Jia

**Affiliations:** a Department of Neurology, The First Affiliated Hospital of Zhengzhou University, Zhengzhou, China; b Department of transfusion, The Second Affiliated Hospital of Zhengzhou University, Zhengzhou, China

**Keywords:** Erythrocytes, Multiple sclerosis, Neuroinflammation, Oxidized mitochondrial DNA, TLR9

## Abstract

Multiple sclerosis (MS), an autoimmune disorder of the central nervous system(CNS), is charactered by neuroinflammation and neurodegeneration. Red blood cells (RBCs) are the most abundant cells in circulation, yet their immunological roles remain poorly understood, especially in neuroimmunology. Here, we explored the role and mechanism of erythrocytes in linking CNS pathology to systemic inflammation in MS, by combining clinical epidemiology and experimental model validation. In UK Biobank (1,056 MS patients; 501,314 controls), MS patients exhibited significantly altered erythrocyte function. Notably, lower baseline erythrocyte counts were inversely associated with MS incidence. Our clinical cohort of 110 relapsing-remitting MS (RRMS) patients revealed progressive declines in RBC parameters (RBC counts, hemoglobin, hematocrit) which associated with disease duration and worse EDSS scores. In RRMS patients, RBCs proteomics and flow cytometry revealed TLR9 membrane translocation and CD47 downregulation. Ox-mtDNA-bound erythrocytes were more phagocytosed by macrophages, which subsequently triggered inflammatory activation and type I interferon responses. These findings were corroborated in the EAE model, where CNS-derived ox-mtDNA was found bound to erythrocyte TLR9, accompanied by compensatory erythropoiesis and interferon-β-driven inflammation. Treatment with the mitochondrial-targeted antioxidant MitoQ effectively normalized erythrocyte TLR9/CD47 expression, reduced ox-mtDNA release, and alleviated neuroinflammation. Collectively, our work defines a novel erythrocyte-TLR9-ox-mtDNA axis that links CNS mitochondrial damage to peripheral inflammation in MS. By integrating clinical cohort data with proteomics and experimental models, we establish erythrocyte pathology as a central feature of MS and highlight the therapeutic potential of targeting mitochondrial dysfunction to disrupt this cascade.

## Graphical abstract

**Figure:**
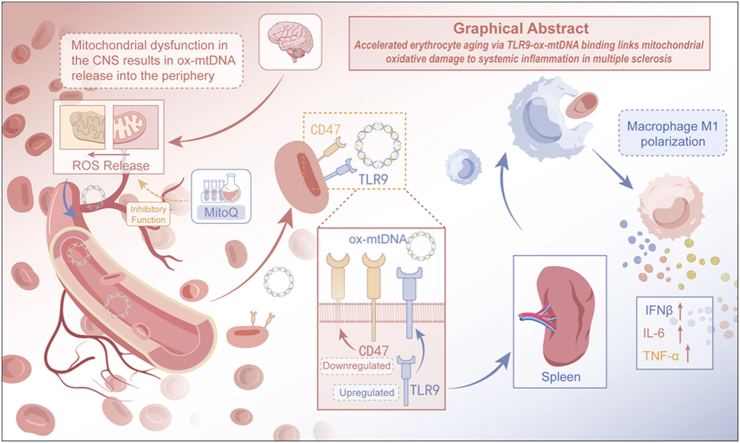

## Introduction

The interplay between systemic immunity and central nervous system (CNS) pathology is a cornerstone of neuroinflammatory disorders like multiple sclerosis (MS), where peripheral immune activation drives disease progression and neural injury [1,2]. Over the past decade, a 30 % rise in MS incidence has compounded the substantial neurological and socioeconomic burden caused by its relapsing-remitting course [3,4]. Although research has heavily focused on adaptive immune cells, the pathophysiology of MS remains incompletely understood. New evidence suggests that in addition to the involvement of the CNS, systemic immune activation is observed in peripheral blood leukocytes during the neuroinflammatory process of MS(5, 6). However, the role of erythrocytes, the most abundant circulating cells, in modulating immune responses and contributing to MS progression has been largely overlooked, creating a critical gap in our knowledge.

Erythrocytes, traditionally viewed as passive oxygen carriers, are now recognized as active participants in innate immunity through cytokine scavenging, complement regulation, and pathogen clearance [7,8]. Recent evidence demonstrates that erythrocytes express functional Toll-like receptors (TLRs), including TLR9, enabling them to interact with damage-associated molecular patterns (DAMPs) such as mitochondrial DNA (mtDNA) [[9], [10], [11]]. Under pathological conditions, oxidative stress and mitochondrial dysfunction drive the release of oxidized mtDNA (ox-mtDNA), which acts as a potent TLR9 ligand triggering proinflammatory cascades [12]. While TLR9 activation in immune cells has been implicated in MS pathogenesis [13], the functional consequences of erythrocyte TLR9 signaling—particularly in response to CNS-derived ox-mtDNA—remain unexplored.

Mitochondrial dysfunction is a hallmark of MS, marked by oxidative damage, impaired respiratory chain activity, and mtDNA mutations in neurons and glial cells [14,15]. Demyelination-induced metabolic enhancement exacerbates mitochondrial damage and ox-mtDNA release, but the consequences of these events are poorly defined [16,17]. Intriguingly, erythrocytes, as a link between the CNS and systemic inflammation, may be involved in the transport of mitochondrial DNA and the regulation of mitochondrial function [18,19]. However, no studies have investigated whether erythrocytes carrying ox-mtDNA contribute to inflammatory activation of macrophages or type I interferon (IFN) signaling in MS, despite the important role of IFN pathways in disease progression.

This study was designed to define the pathogenic role of erythrocytes in multiple sclerosis. We integrated data from the UK Biobank, a clinical cohort of 110 relapsing-remitting MS (RRMS) patients, and the experimental autoimmune encephalomyelitis (EAE) mice model. We propose that CNS mitochondrial dysfunction in MS leads to the release of oxidized mitochondrial DNA (ox-mtDNA) into the circulation. This ox-mtDNA then binds to TLR9 on erythrocytes, accelerating their phagocytic clearance by splenic macrophages and triggering type I interferon-mediated systemic inflammation. To test this hypothesis, we employed the mitochondrial-targeted antioxidant MitoQ. Our results show that MitoQ treatment alleviates oxidative damage in neurons and glia, suppresses neuroinflammation, and reduces circulating ox-mtDNA levels. Consequently, TLR9 expression on erythrocytes is normalized, their clearance by splenic macrophages is reduced, and type I interferon pathway activation is inhibited. By validating this erythrocyte-TLR9-ox-mtDNA axis, we aim to establish erythrocytes as crucial mediators linking CNS damage to peripheral inflammatory, offering new insights into the immunopathology of MS.

## Materials and methods

### Study design and participants

#### Sample size estimation

This prospective cohort study is an observational clinical study conducted from January 2023 to June 2025 at the First and Second Affiliated Hospitals of Zhengzhou University. This study recruited 110 patients with relapsing remitting multiple sclerosis (RRMS) who visited outpatient clinics or neurology wards, and 103 age and gender matched healthy controls. The recruitment of participants will take place from January 2023 to June 2024, and follow-up and data collection will continue until June 2025.

For sample size estimation, based on preliminary data from our institution, the average difference in red blood cell counts between RRMS patients and healthy controls was 0.35 × 10^12^/L, with a standard deviation of 0.55 × 10^12^/L. We calculated that each group of 98 participants would provide 90 % of their energy (two-tailed) at α=0.05. In order to consider the potential for loss of follow-up and missing data, we increased the sample size of RRMS patients to 110.

All patients with MS were diagnosed in accordance with the 2017 McDonald criteria, while those with NMOSD were diagnosed using the 2015 diagnostic criteria established by the International Panel for NMO Diagnosis (IPND) [20,21]. All diagnoses were independently confirmed by two neurologists. Participants provided comprehensive demographic data (including sex and age), disease characteristics (annualized relapse rate, limb weakness, sensory disturbances, visual impairment, and ataxia), as well as erythrocyte and inflammation-related biochemical markers. Each subject underwent brain and spinal cord MRI, with lesion counts quantified to assess disease severity. Neurological disability was evaluated using the Expanded Disability Status Scale (EDSS). Written informed consent was obtained from all study participants.

Inclusion criteria comprised: [1] age 18-65 years; [2] Meets diagnostic criteria and has been diagnosed with MS or NMOSD by two neurologists; and [3] willingness of patients to provide informed consent for blood testing. Exclusion criteria included: [1] comorbid immune-related diseases; [2] hematologic disorders (e.g., aplastic anemia, myelodysplastic syndrome, or hematologic malignancies); [3] pregnancy or lactation; [4] incomplete clinical data; and [5] antibiotic use within the preceding three months. Conduct a telephone follow-up every 6 months for all RRMS patients, including assessment of disease recurrence and severity (EDSS).

The study was reviewed and approved by the Ethics Review Committees of the First and Second Affiliated Hospitals of Zhengzhou University (2023-KY-1303-003, KY2025162).

Peripheral blood samples from all participants were processed using a standardized protocol [10]. All blood specimens from MS patients treated steroid were collected at least 4 weeks after the completion of steroid pulse therapy. Briefly, whole blood was centrifuged at 500×*g* for 10 minutes at 4 °C to separate plasma and cellular components. The erythrocyte pellet was resuspended in PBS and passed through a leukocyte reduction filter (Acrodisc® White Blood Cell Syringe Filter, Pall Medical). The filtered suspension was centrifuged at 800×*g* for 5 min to pellet the purified erythrocytes. Both erythrocyte and plasma samples were stored at −80 °C until further analysis.

### Variables

Potential confounders identified a priori included age, sex, renal function (serum creatinine), liver function (ALT, AST), and blood coagulation condition (APTT, INR). Effect modifiers considered were disease-modifying therapies, steroid use, and comorbidities affecting erythrocyte turnover.

### Data sources/measurement

The clinical erythrocytes parameters (erythrocytes count, hemoglobin, hematocrit, etc.) of all subjects recruited in the prospective clinical cohort of this study were measured by fully automated blood analyzers at the First and Second Affiliated Hospital of Zhengzhou University . The severity of the disease was evaluated using the EDSS by neurologists at the First Affiliated Hospital of Zhengzhou University. The red blood cell flow cytometry parameters (CD47, TLR9 expression), plasma inflammatory cytokine levels of the subjects were measured using commercial assay kits, and erythrocytes cytoplasmic protein was quantitatively analyzed using 4D-DIA proteomics.

### Bias

To mitigate selection bias, consecutive eligible RRMS patients were recruited during routine clinical visits, and healthy controls were matched for age and sex. Measurement bias was addressed by standardizing all laboratory protocols, with blood sample processing within 2 h of collection and batch analysis of subjects. blinding was implemented for key assessments: flow cytometry operators were blinded to group assignment, and neurologists performing EDSS evaluations were blinded to laboratory results. Confounding bias was controlled through multivariate regression models adjusting for age, sex, renal function, and disease-modifying therapies.

### Quantitative variables

Quantitative variables were analyzed primarily as continuous measures to preserve statistical power, with categorization applied when clinically meaningful. Disease duration was stratified at 5 years, a key time point with disability progression in RRMS. The EDSS score was dichotomized at >3, a standard threshold distinguishing significant neurological impairment. All categorization criteria were pre-specified before analysis, using established clinical standards where available or data-driven thresholds validated within the cohort. This dual approach maintained analytical sensitivity while ensuring clinical relevance and interpretability of findings across different disability states and disease phases.

### GEO database and UK biomark database

According to the previous method, mitochondrial metabolism related genes were extracted from the GeneCards database (https://www.genecards.org/) using the keyword "mitochondrial metabolism". Transcriptome data from peripheral blood mononuclear cells (PBMC) of MS patients and healthy controls (HCs) were acquired from the GSE136411 dataset in the Gene Expression Omnibus (GEO) database (https://www.ncbi.nlm.nih.gov/geo/; accessed on March 2024). Differentially expressed genes (DEGs) were identified via limma package. Gene ontology (GO) enrichment analysis was performed to uncover biological processes associated with DEGs, which was realized by BioEnricher package.

The UK Biobank (UKB) is a large, detailed, prospective, population-based study of over 500,000 participants aged 40-69 years. The cohort includes extensive phenotypic and genotypic details of the participants, including questionnaires, physical measurements, sample analysis and genome-wide genotyping data; we used phenotypic and genetic data from 477,069 participants in the UK Biobank, excluding withdrawn participants and individuals with mismatched information. Quality control of the clinical data was performed centrally by the UK Biobank. More information about the cohort can be found on its official website (https://www.ukbiobank.ac.uk/; accessed on April 2024). Cox regression modeling to estimate the association between red blood cell count and risk of developing multiple sclerosis in participants of the UK Biobanking Study.

### Animals

**Groups:** A total of 90 female C57BL/6J mice (6-week-old) were randomly assigned into three experimental groups (n=30 per group) using a random number table method: [1] the EAE group; [2] the EAE + MitoQ group; and [3] the Sham group. EAE mice were immunized with MOG peptide and PTX on day 0, followed by a booster injection of PTX on day 2. The EAE + MitoQ group received additional MitoQ treatment administered every other day throughout the study period. Sham mice were injected with an equivalent volume of PBS on both day 0 and day 2. The sample size is determined based on the needs of subsequent pathological experiments to ensure sufficient statistical capacity.

### EAE mice model

The EAE mice model was established following previously described methods [22,23]. SPF-grade 6-week-old female C57BL/6J mice were procured from the BEIJING HFK BIOSCIENCE Co.,LTD with an average body weight of 18±2 g. All mice were allowed to freely come into contact with standard rodent food and high-pressure water for 1 week under standard laboratory conditions (12/12 hour light/dark cycle, ambient temperature of 22 ± 1 °C) to adapt to the environment. The induction of the EAE model involved immunization with myelin oligodendrocyte glycoprotein 35-55 (MOG35-55) (sequence MEVGWYRSPFSRVVHLYRNGK) in conjunction with complete Freund's adjuvant (CFA). A total of 200 μg of MOG_35-55_ per mice was emulsified in an equal volume of Complete Freund's Adjuvant (CFA) containing 5 mg/ml of Mycobacterium tuberculosis H37Ra. The emulsion was administered via subcutaneous injection at multiple sites across both sides of each mice back. Additionally, EAE mice received intraperitoneal injections of 200 ng of pertussis toxin (PTX) (P7208, Sigma) on days 0 and 2 postinoculation. The sham group received subcutaneous injections of an equal volume of PBS. All the injection and scoring procedures were conducted in a double-blinded manner, and a scoring system similar to the standard 0–5 scale was used to evaluate the clinical signs of EAE [24,25]. For ethical considerations, mice that reached a score of 4 or 5 for two consecutive days were euthanized, and their experimental data were retained. All animal experimental sections were reviewed by the Laboratory Animal Ethics Committee (Approval No. V3A02024113003) in February 2024.

### Intraperitoneal Administration of MitoQ

This study adopted a preventative administration paradigm. Mitoquinone (MitoQ) (mitochondria-targeted ubiquinone) was administered via intraperitoneal injection to EAE mice [26,27]. Prior to administration, MitoQ (HY-100116A, MCE) was supplied as a 10 mg/mL stock solution in sterile dimethyl sulfoxide (DMSO), which was diluted 20-fold in sterile 0.9 % normal saline to a final MitoQ concentration of 0.5 mg/mL, resulting in a final DMSO concentration of 5 % (v/v) in the injection solution. EAE mice were treated with MitoQ at a dose of 5 mg/kg body weight every other day, starting from the day of MOG35-55 induction (day 0). Vehicle control animals received an equivalent volume of sterile 0.9 % normal saline containing 5 % (v/v) DMSO following the same administration schedule. No significant differences were observed between the vehicle-treated and EAE groups in terms of clinical disease scores, demyelination levels, and mitochondrial functional damage.

### Organizational processing

Euthanasia was performed on EAE mice with pentobarbital sodium at 0d, 7d, 14d, 21d, and 28d after induction, and samples were collected according to standard procedures [23,28]. Peripheral blood samples were collected from the orbital area of EAE mice and centrifuged at 500×*g* for 5 min to separate erythrocytes from plasma and leukocytes [11]. The erythrocyte sediment was mixed with PBS, and purified erythrocytes were obtained by passing the sediment through a leukocyte reduction filter (Acrodisc® White Blood Cell Syringe Filter, Pall Medical) to remove leukocyte contamination interference. This filtration method was verified to preserve the expression of erythrocyte surface receptors. Brain, spleen, and femur tissues were collected following cardiac perfusion with ice-cold PBS. The whole spleen was weighed, and the femur was decalcified. The brain and spleen tissues were paraffin-embedded and sectioned into 8-μm slices.

### THP-1 cell culture and coculture with erythrocytes

A THP-1 cell line (STCC10907P) was procured from Service Biologicals and cultured in RPMI 1640 medium supplemented with 10 % FBS and 2 mmol/L l-glutamine at a concentration of 2 × 10^5^ cells/ml. Upon reaching the appropriate density, THP-1 cells (2 × 10^5^/ml) were induced to differentiate into macrophages via the administration of 200 nM phospho-12-myristate-13-acetate (PMA; Sigma‒Aldrich Corp.) for 48 h [29,30]. Following removal of the PMA-containing medium, the differentiated macrophages were used for co-culture experiments. Equal volumes of purified erythrocytes from RRMS patients or HC were added to the macrophage cultures at a target erythrocyte-to-macrophage ratio of 10:1 and coincubated for 3 h in fresh RPMI 1640 (10 % FBS+1 % l-glutamine) medium. Controls were treated with equivalent volumes of erythrocytes from individuals in the HC group and coincubated. The primary outcome indicators for this assay were the erythrocyte phagocytosis rate by macrophages and the subsequent inflammatory activation of macrophages, specifically the induction of interferon-β (IFN-β).

### 4D-DIA Quantitative Proteomics

Fresh whole blood collected from the subjects was centrifuged at 500×*g* for 10 min at 4 °C to separate the plasma and buffy coat. The resulting erythrocyte pellet was resuspended in PBS and passed through a leukoreduction filter (Acrodisc White Blood Cell Syringe Filter, Pall Medical). The filtered suspension was then centrifuged at 800×*g* for 5 minutes, and the supernatant was discarded to obtain purified erythrocytes. The purified erythrocytes were subjected to hypotonic lysis using 0.83 % NH_4_Cl to remove cellular membranes. The lysate was then centrifuged at 1,500×*g* for 5 minutes to obtain a purified cytoplasmic fraction (supernatant). This cytoplasmic fraction was subsequently lysed in 8 M urea/ 2 % SDS buffer supplemented with protease inhibitors (Roche), and the protein concentration was quantified using the BCA assay (Pierce). For TMTpro 16-plex labeling, 100 μg protein per sample was reduced (10 mM DTT, 30 min, 55°C), alkylated (50 mM iodoacetamide, 15 min dark), and digested with Trypsin Gold (Promega, 1:50 w/w, 37 °C, 16 h). Peptides were labeled per manufacturer’s instructions (Thermo Fisher), pooled, and desalted using C18 solid-phase extraction. 4D-LC-MS/MS analysis was performed on a nanoElute UHPLC system (Bruker) coupled to a timsTOF Pro 2 mass spectrometer [31,32]. Peptides were separated on a 25-cm C18 column (1.9 μm, 120 Å) with a 120-min gradient (6–30 % ACN/0.1 % FA) at 300 nL/min. Data-independent acquisition (DIA) employed diaPASEF mode with ion mobility (1/K0 range: 0.6–1.4 V s/cm^2^), *m/z* 100–1700, and 10 MS/MS scans/cycle. The primary outcome indicators were the relative abundance of erythrocyte cytoplasmic proteins and the identification of differentially expressed proteins (DEPs) between RRMS patients and healthy controls. Protein identification and quantification were performed against the UniProt human reference proteome database. The quantification of proteins was based on the extracted ion chromatograms of their constituent peptides, with the relative abundance of each protein calculated from the median normalized intensity of its uniquely identifying peptides across all samples. DEPs were defined as those exhibiting a |log_2_(fold change)| > 0.58 (equivalent to a 1.5-fold change) and an adjusted p-value < 0.05. Functional classification analysis, including Gene Ontology (GO) secondary classification and KEGG pathway enrichment for the DEPs, was performed. Use Fisher's exact test method to perform GO and KEGG enrichment analysis on the DEPs between the two groups.

### Statistical analysis

All statistical computations were performed using SPSS software (version 21.0; IBM SPSS, Chicago, IL, USA), with graphical representations created in GraphPad Prism 9. For the primary analysis of quantitative data derived from clinical assessments, flow cytometry, ELISA, PCR, and osmotic fragility assays, we first examined all variables for normality using Shapiro-Wilk tests. Data conforming to normal distribution are presented as mean values ± standard deviations, while non-normally distributed variables are reported as medians with interquartile ranges (IQRs). For variables conforming to normal distribution, independent samples t-tests were employed to analyze differences between the two groups. Clinical scores among multiple groups (Sham, EAE, and EAE + MitoQ) were compared using the Friedman test, a nonparametric alternative to two-way analysis of variance. To address potential confounding factors in our clinical cohort analysis, we implemented multiple strategies. For the comparison of erythrocyte parameters between RRMS patients and healthy controls, we constructed multivariable linear regression models adjusting for age, sex, and key laboratory parameters that might influence erythrocyte indices. This approach allowed us to isolate the specific association between MS status and erythrocyte abnormalities. Subgroup analyses were pre-specified in our analytical plan to explore potential effect modification. We examined subgroups defined by disease duration (dichotomized at 5 years) and disability level (EDSS score stratified at 3). To formally test for interactions between these subgrouping variables and our primary outcomes, we introduced interaction terms into our regression models and assessed their statistical significance using likelihood ratio tests. Categorical data were expressed as the number of cases and were compared using either the chi-square test or its corrected version. For statistical assessments involving more than two groups, one-way ANOVA was applied for multiple comparisons. Correlation analyses employed Spearman's rank correlation coefficient to explore associations between erythrocyte-related indices and clinical variables including disease severity, duration, and annual recurrence rate. A two-sided P-value <0.05 was considered statistically significant for all analyses.

## Results

### UK Biobank Reveals Distinct Erythrocyte Abnormalities in MS Patients

**Figure 1:**
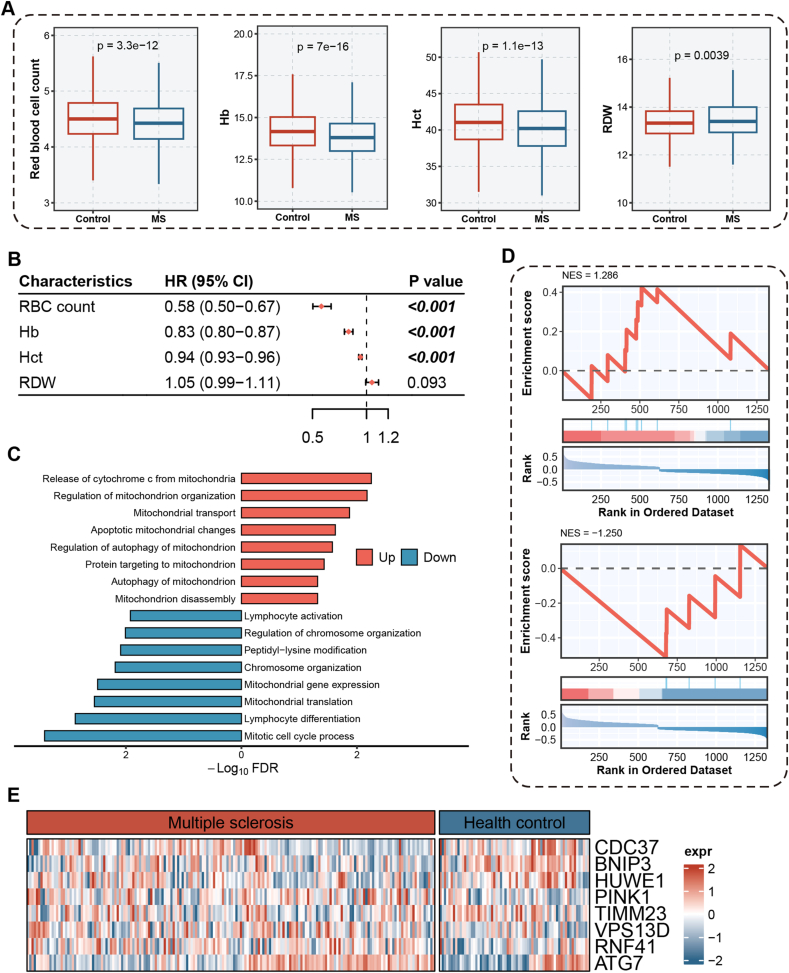
**UKB database to screen RRMS patients for erythrocyte alterations. (A)** The results of the cross-sectional study showed a decrease in erythrocyte count, hemoglobin content, erythrocyte pressure volume, and an increase in erythrocyte distribution width in patients with MS compared to HC. **(B)** 476,009 participants who did not have MS at baseline were followed for a median follow-up of 13.78 years, and Cox regression analyses showed that higher erythrocyte count were associated with a lower prevalence of MS. **(C,D)** A search of the GEO database revealed that mRNA expression in pathways associated with mitochondrial autophagy, apoptosis, and catabolism was dysregulated in the peripheral blood of patients with multiple sclerosis compared with healthy controls. **(E)** In MS patients, 8 genes, including *PINK1*, *HUWE1*, and *ATG7*, were significantly different.

Our comprehensive analysis of hematological parameters in patients with MS revealed consistent alterations in erythrocyte profiles across multiple independent datasets. In a cross-sectional analysis of the UK Biobank (UKB) cohort at baseline, which initially included 502,370 participants—1,056 with pre-existing MS and 501,314 without—we excluded 24,328 individuals with missing data on erythrocyte-related variables. The final analytical sample comprised 478,042 participants (973 with MS and 477,069 without). The analyzed cohort had a mean age of 56.55 (±8.09) years, was 45.9 % male, and 90.98 % White, with a mean BMI of 27.41 (±4.78). Within this sample, individuals with MS exhibited significantly lower erythrocyte counts (4.42 ± 0.41 vs. 4.52 ± 0.42, *P*<0.001), hemoglobin levels (13.86 ± 1.25 vs. 14.18 ± 1.25, *P*<0.001), and hematocrit (40.26 ± 3.53 vs. 41.09 ± 3.55, *P*<0.001), along with elevated red cell distribution width (13.66 ± 1.36 vs. 13.49 ± 0.99, *P*=0.0039) (Fig. 1a).

In a subsequent prospective analysis, after excluding 24,328 participants with missing data on key erythrocyte measures (specifically, 24,325 missing RBC count/Hb/Hct and 24,327 missing RDW), we retained 478,042 participants. From these, we further excluded 973 individuals diagnosed with MS prior to baseline, resulting in a final cohort of 477,069 disease-free participants with complete baseline data. Over a median follow-up of 13.8 years (maximum 16 years), 1,060 incident MS cases were identified, while 476,009 participants remained free of MS. Notably, higher baseline erythrocyte counts were significantly associated with a reduced risk of developing MS (Fig. 1b), suggesting a potential protective role of erythrocyte abundance against the onset of MS.

**Figure:**
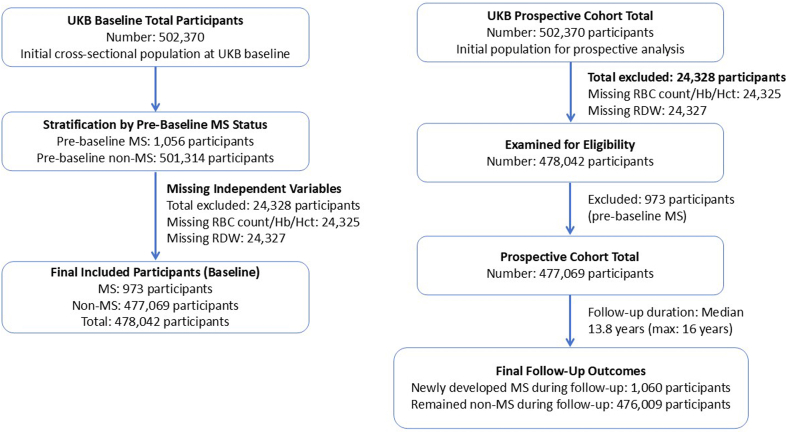

### Erythrocyte Abnormalities in RRMS Patients Associate with Disease Progression and Disability

**Figure 2:**
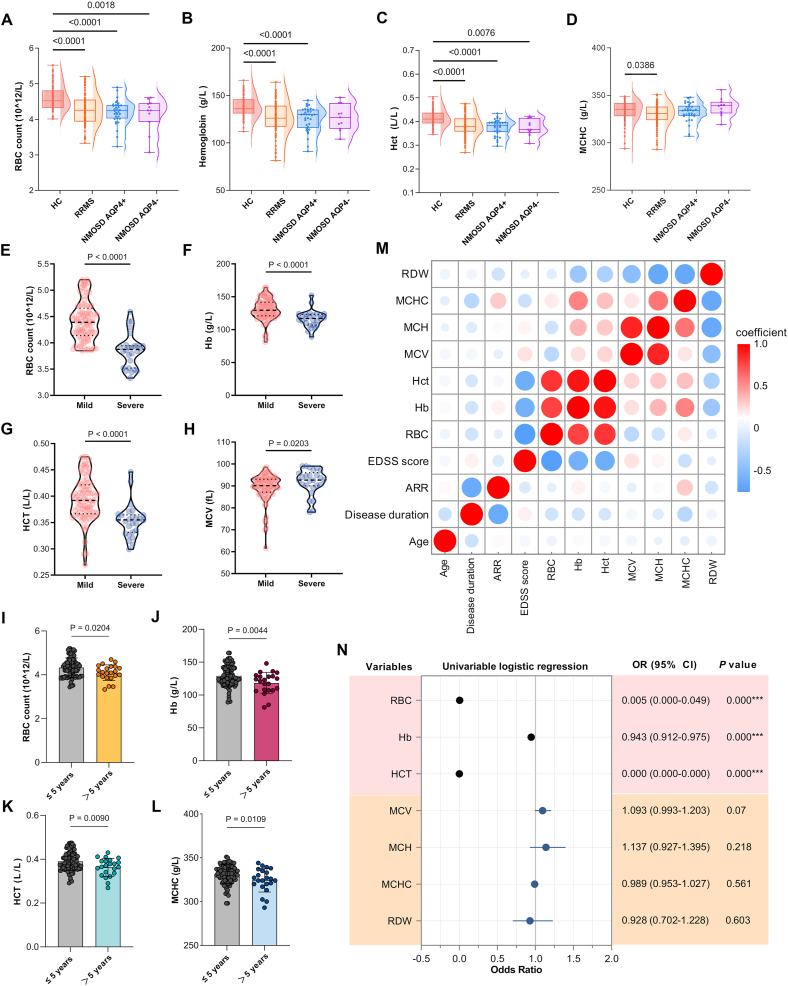
**Clinical data, correlation analysis, and risk factor assessment. (A-D)** Quantitative comparison of RBC count, Hb level, Hct, and MCHC among RRMS patients, NMOSD patients, and HC showed that RRMS patients had significantly lower RBC count, Hb level, Hct, and MCHC than HC, while NMOSD patients only displayed a decreasing trend. **(E-H)** Subgroup analysis of RRMS patients stratified by EDSS score revealed that patients with higher EDSS scores had significantly reduced RBC count, Hb level, Hct, and MCV. **(I-L)** When comparing RRMS patients grouped by disease duration (5-year cutoff), those with longer disease duration showed significantly decreased RBC count, Hb level, and Hct but increased MCHC. **(M)** Spearman correlation analysis demonstrated associations between RBC-related parameters (RBC count, Hb, Hct, MCV, MCH, MCHC, and RDW) and demographic characteristics in RRMS patients. **(N)** Univariate logistic regression analysis identified RBC count, Hb level, and Hct as independent risk factors for RRMS. ∗∗∗ as *P*<0.001.

The clinical cohort study included 110 RRMS patients (mean age 40.49±13.11 years; 74.5 % female) and 103 age- and sex-matched healthy controls (mean age 42.97±11.12 years; 72.8 % female), with no significant demographic differences between groups (*P*>0.05) (Table 1). The RRMS cohort exhibited diverse clinical presentations, 44.5 % with limb weakness, 53.6 % with sensory abnormalities, 34.5 % with visual disturbances, and 28.2 % with ataxia. Disease characteristics included a median disease duration of 24 months (IQR 2.75-50), annualized relapse rate (ARR) of 1.0 (IQR 0.5-7.5), median lesion count of 5 (IQR 3-7) on 3.0T MRI, and median EDSS score of 2.0 (IQR 1.0-3.0) (Table 1). Comparative analysis demonstrated significantly reduced erythrocyte counts (median 4.25 vs 4.52 × 10^12^/L, *P*<0.001), hemoglobin levels (mean 127.08 vs 138.16 g/L, *P*<0.001), and hematocrit (mean 0.384 vs 0.414 L/L, *P*<0.001) in RRMS patients versus controls, along with lower mean corpuscular hemoglobin concentration (MCHC) (median 331 vs 335 g/L, *P*<0.05), while other erythrocyte indices showed no significant differences (Table 1; Fig. 2a-d; sFig. 1a, b). Although NMOSD patients demonstrated a decreasing trend in RBC parameters, none reached statistical significance compared to HC group.

**Table 1:** General information and clinical indicators of the participants

	RRMS (n=110)	NC (n=103)	NMOSD(AQP-4+) (n=33)	NMOSD(AQP-4-) (n=11)	*P* (RRMS and NC)
**General Information**					
Sex(M:F)	82:28	75:28	3:30	2:9	0.876
Age, years	40.49±13.11	42.97±11.12	44.66±14.24	50.90±19.41	0.138
months of oneset	24(2.75-50)	NA			
Annualized relapse rate (ARR),median(IQR)	1.0(0.5-7.5)	NA			
MRI,Number of lesions(IQR)	5(3-7)	NA			
EDSS score	2.0(1.0-3.0)	NA			
**Treatment**					
Steroids,n%	72(65.5 %)	NA			
Fingolimod,n%	3(2.7 %)	NA			
Siponimod,n%	4(3.6 %)	NA			
Ofatumumab,n%	8(7.3 %)	NA			
**Clinical symptoms**					
weakness,n%	49(44.5 %)	NA			
sensory abnormality,n%	59(53.6 %)	NA			
vision problem,n%	38(34.5 %)	NA			
ataxia,n%	31(28.1 %)	NA			
**Laboratory test results**					
RBC,10ˆ12/L	4.25(3.93-4.55)	4.52(4.32-4.81)	4.24(4.02-4.39)	4.25(3.93-4.45)	<0.001∗∗∗
WBC,10ˆ9/L	5.92(4.63-7.48)	6.14(5.16-7.55)	6.87(4.67-11.07)	6.43(5.81-11.38)	0.361
PLT,10ˆ9/L	230(182.75-279.25)	243(203-283)	216(177-287.5)	243(195-279)	0.089
Hb,g/L	127.08±16.54	138.16±11.43	125.17±12.84	126.69±13.69	<0.001∗∗∗
RDW,median(IQR),%	13(12.5-13.9)	13(12.6-13.4)	13.8(12.75-17.55)	13.5(12.3-16.81)	0.824
Hct,L/L	0.384±0.043	0.414±0.033	0.37±0.03	0.37±0.03	<0.001∗∗∗
MCV,fL	90.9(87.75-93.52)	90.4(88.4-92.7)	90.30(86.85-93.80)	90.60(83.10-97.70)	0.455
MCH,pg	30.3(29-31.4)	30.3(29.3-31.1)	30.40(28.80-31.06)	31.1(28.10-32.20)	0.728
MCHC,g/L	331(324-338.02)	335(328-342)	334.00(328.50-339.00)	339.00(331.00-343.00)	<0.05∗
N%,%	62.2(56.67-71.55)	60.5(56.9-64.0)	68.1(57.35-82.75)	71.6(60.9-73.0)	0.089
L%,%	27.8(18.17-34.42)	30.4(26.3-34.0)	24.7(14.7-33.25)	24.2(20.2-29.7)	<0.05∗
TB,μmol/L	9.08(6.46-12.3)	8.2(6.5-11.3)	8.00(6.35-10.10)	8.60(6.80-11.70)	0.398
CB,μmol/L	3.7(2.59-5.00)	3.6(2.8-4.8)	3.40(3.10-4.30)	4.40(2.80-5.70)	0.833
ALT,U/L	15(10-23)	18(13-27)	16.00(10.00-35.00)	17.00(14.00-39.00)	0.066
AST,U/L	16.5(13-21)	17(14-23)	15.00(13.00-19.50)	17.00(12.00-23.00)	0.181
GGT,U/L	16.0(11.0-25.0)	22.0(12.0-28.0)	21.00(14.00-55.00)	18.00(14.00-46.00)	0.052
TP,g/L	65.46±5.66	66.84±5.56	65.05±6.03	66.95±7.56	0.074
ALB,g/L	41.5(39.7-43.82)	42.3(40.4-44.6)	41.00(37.75-42.85)	38.90(35.60-44.20)	0.14
Cr,μmol/L	54.0(48.0-64.0)	57.0(52.0-63.0)	53.00(45.00-59.50)	56.00(46.00-60.00)	0.151
BUN,mmol/L	4.55(3.8-5.3)	4.4(3.8-5.5)	5.00(3.38-6.44)	4.70(3.20-7.61)	0.711
UA,μmol/L	227.0(196.75-307.75)	249.0(214.0-280.0)	229.0(184.5-244.50)	194.00(166.00-212.00)	0.832
PT,s	11.2(10.67-11.72)	11.1(10.6-11.7)	11.1(10.3-11.95)	11.1(10.9-12.0)	0.654
INR	1.02(0.96-1.06)	1.01(0.96-1.05)	1.01(0.94-1.08)	1.01(0.99-1.09)	0.588
APTT,s	29.06±3.81	28.53±2.70	27.42±3.05	26.59±2.78	0.241
TT,s	15.93±1.85	15.87±1.71	16.1(14.75-17.05)	15.2(14.7-16.2)	0.814
D-Dimer,mg/L	0.15(0.07-0.19)	0.1(0.05-0.19)	0.19(0.05-0.33)	0.12(0.07-0.22)	0.132

Note: This table summarizes the baseline characteristics, clinical presentations, and laboratory findings across study groups, including relapsing-remitting multiple sclerosis (RRMS) patients, neuromyelitis optica spectrum disorder (NMOSD) patients (both aquaporin-4 antibody-positive and negative), and neurologically healthy controls (NC). It highlights the specific erythrocytic abnormalities—such as reduced red blood cell count, hemoglobin, and hematocrit—that are distinctive to RRMS compared to other demyelinating disorders and controls, underscoring the unique role of erythrocytes in MS pathophysiology. ALB, Albumin; ALT, Alanine Aminotransferase; APTT, Activated Partial Thromboplastin Time; ARR, Annualized Relapse Rate; AST, Aspartate Aminotransferase; BUN, Blood Urea Nitrogen; CB, Conjugated Bilirubin; Cr, Creatinine; EDSS, Expanded Disability Status Scale; GGT, Gamma-Glutamyl Transferase; Hb, Hemoglobin; Hct, Hematocrit; INR, International Normalized Ratio; IQR, Interquartile Range; L%, Lymphocyte Percentage; MCH, Mean Corpuscular Hemoglobin; MCHC, Mean Corpuscular Hemoglobin Concentration; MCV, Mean Corpuscular Volume; MRI, Magnetic Resonance Imaging; N%, Neutrophil Percentage; PLT, Platelet Count; PT, Prothrombin Time; RBC, Red Blood Cell Count; RDW, Red Cell Distribution Width; TB, Total Bilirubin; TP, Total Protein; TT, Thrombin Time; UA, Uric Acid; WBC, White Blood Cell Count.

Stratified analysis by disease duration revealed more pronounced erythrocyte abnormalities in patients with ≥5 years disease duration compared to those with <5 years duration, including significantly lower erythrocyte counts (mean 4.09 vs 4.33 × 1012/L, *P*<0.05), hemoglobin levels (mean 118.2 vs 129.3 g/L, *P*<0.01), hematocrit (mean 0.363 vs 0.390 L/L, *P*<0.01), and MCHC (median 325 vs 332 g/L, *P*<0.05) (Fig 2i-l; sTable 2). Disability-stratified analysis (EDSS ≤3 vs >3) showed that patients with severe neurological impairment had more marked erythrocyte reductions (median 3.87 vs 4.39 × 1012/L, *P*<0.0001) and hemoglobin decreases (median 117 vs 128.8 g/L, *P*<0.0001) compared to mildly impaired patients, along with elevated mean corpuscular volume (MCV) (median 92.67 vs 90.14 fL, *P*<0.05) and mean corpuscular hemoglobin (MCH) (median 31.1 vs 30.1 pg, *P*<0.05) (Fig. 2e-h; sTable 3; sFig. 1h).

Further Spearman correlation analysis was performed to evaluate the relationship between erythrocyte parameters and both disease duration and clinical severity in RRMS patients. Among the 110 RRMS cases, significant negative correlations were observed between EDSS scores and three hematological indices: RBC count (r=-0.7088, *P*<0.0001), hemoglobin (r=-0.597, *P*<0.01), and hematocrit (r=-0.626, *P*<0.01) (Fig. 2m). This inverse correlation pattern indicates that higher values of these erythrocyte parameters were associated with milder neurological impairment. However, no statistically significant associations were detected between these hematological markers and either disease duration or annualized relapse rate in the RRMS cohort (Fig. 2m; sFig. 1d-f).

Univariate logistic regression analysis was performed to evaluate whether erythrocyte parameters could serve as risk factors for MS. The results demonstrated that decreased RBC count, Hb, and Hct were all significant independent risk factors for MS development (all *P*<0.0001) (Fig. 2n). The diagnostic performance of RBC count for identifying severe neurological impairment in RRMS patients was evaluated using receiver operating characteristic (ROC) curve analysis. The analysis demonstrated excellent discriminative ability, with an area under the curve (AUC) of 0.870 (95 % CI: 0.788-0.952; *P*<0.001). At the optimal cutoff value of 3.965 × 10^12^/L, RBC count showed balanced sensitivity and specificity of 86.9 % and 80.8 %, respectively, for detecting severe neurological disability in this patient population (sFig. 1c).

Additionally, we collected demographic characteristics and clinical biochemical parameters from 31 patients with peripheral immune-mediated demyelinating disorders (including 24 myasthenia gravis [MG] patients and 7 chronic inflammatory demyelinating polyradiculoneuropathy [CIDP] patients), 21 central nervous system infection patients, and 8 myelin oligodendrocyte glycoprotein antibody-associated disease (MOGAD) patients. Notably, erythrocyte-related parameters (RBC, Hb, Hct, MCV, MCH, MCHC) in these control groups showed no significant differences compared with healthy controls (sTable 1), further confirming the specificity of erythrocyte abnormalities observed in MS patients.

### Erythrocyte molecular pathology and TLR9 dysregulation in RRMS

**Figure 3:**
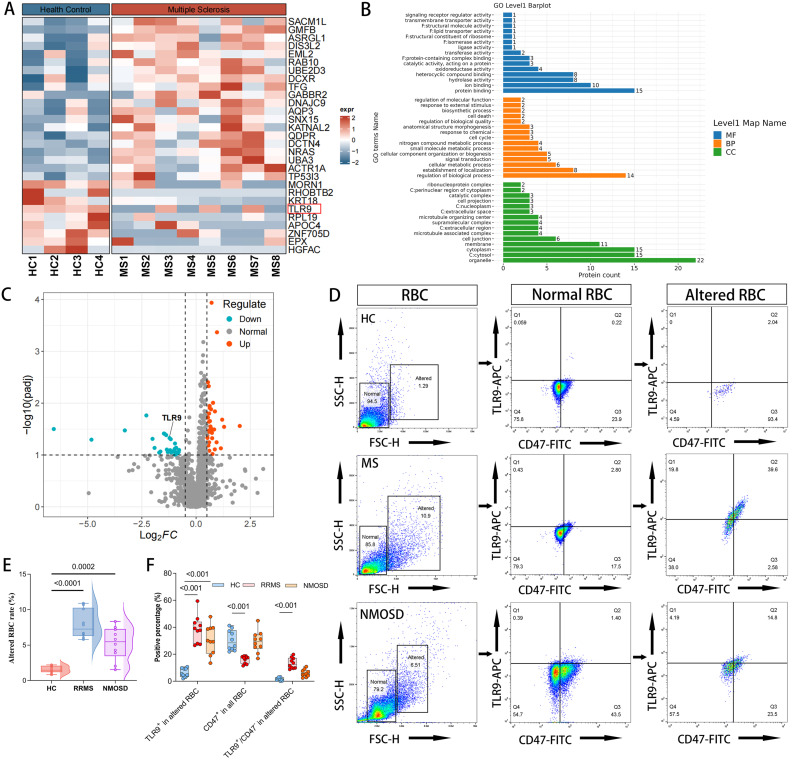
**Differential erythrocyte cytoplasmic and surface proteins in all subjects. (A, C)** Heatmap and volcano plot display the proteomic profiling results of 29 differentially expressed proteins in erythrocyte cytoplasm from 8 RRMS patients and 4 healthy controls. TLR9 levels in erythrocyte cytoplasm were significantly decreased in RRMS patients compared with HC. **(B)** Bar graphs represent GO enrichment analysis in proteomics of RRMS patients vs. healthy controls. The differentially expressed proteins were primarily enriched in signal transduction (GO:0007165), regulation of biological processes (GO:0050789), and protein binding (GO:0005515). **(D-F)** Flow cytometry analysis revealed a significantly increased proportion of deformed erythrocytes in patients with RRMS and NMOSD compared to HC. Additionally, RRMS patients exhibited a decrease in the proportion of CD47-positive erythrocytes and an increase in TLR9-positive erythrocytes on the cell surface. Each group n=10 samples.

After observing reduced RBC counts in RRMS patients, we attempted to determine if their erythrocytes also showed pathological molecular changes. We first compared the cytoplasmic proteomes of erythrocytes from RRMS patients and healthy controls. This identified 29 differentially expressed proteins (Fig. 3a, c). Gene ontology enrichment analysis demonstrated that these proteins were obviously associated with three key biological functions: signal transduction (GO:0007165), regulation of biological processes (GO:0050789), and protein binding (GO:0005515) (Fig. 3b).

Among them, TLR9 caught our attention due to its known role in erythrocyte-mediated immunoregulation TLR9 is a key sensor for self- and pathogen-derived nucleic acids, driving inflammation through cytokine production and immune cell activation. This mechanism is also implicated in how erythrocytes participate in immune regulation during sepsis and pneumonia [11,33]. Intriguingly, we found its expression was significantly lower in the cytosol of RRMS erythrocytes compared to healthy controls (*P*=0.0472). This discovery suggests a potential disruption of the immune regulated TLR9 pathway in RRMS.

Whether the reduced intracellular expression of TLR9 in erythrocytes is associated with vesicular trafficking remains unclear [11,34]. To investigate this, we employed flow cytometry to analyze the expression of erythrocyte membrane surface proteins and morphological changes in peripheral blood samples from patients with RRMS, NMOSD, and HC. Compared to healthy controls, the proportion of morphologically altered erythrocytes was significantly elevated in RRMS patients (*P*<0.0001), while NMOSD patients showed only a modest increase (*P*=0.0002) (Fig. 3d, e). Notably, TLR9 expression was markedly upregulated in the membranes of these deformed erythrocytes in both RRMS and NMOSD groups (*P*<0.001) (Fig. 3d, f). However, the mechanistic link between TLR9 overexpression on erythrocyte membranes and erythrocyte survival remains elusive.

**Figure 4:**
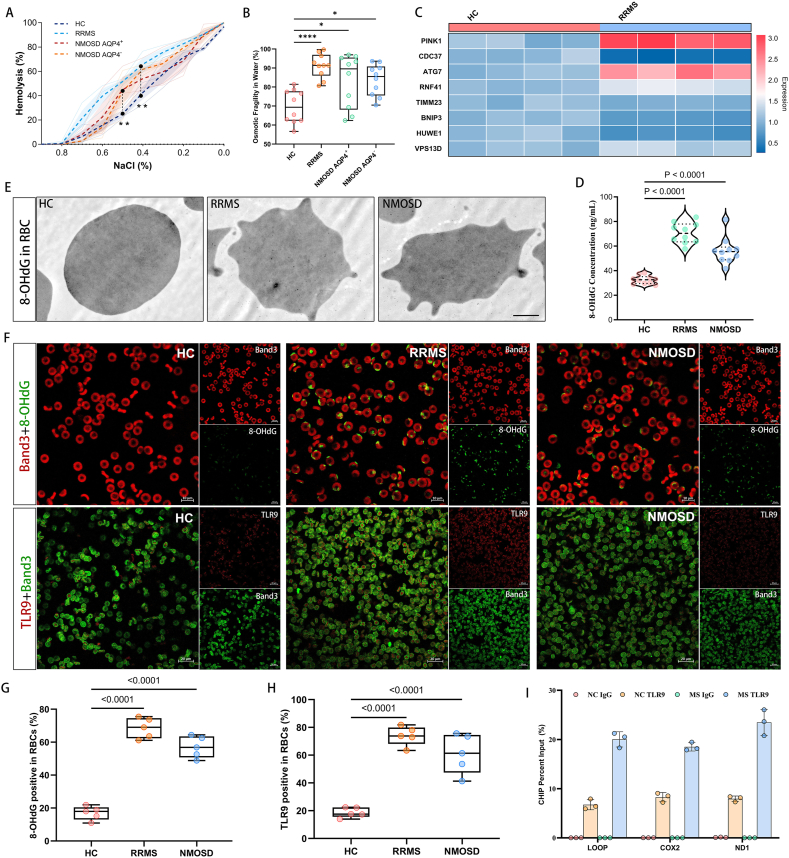
**Altered erythrocyte pathology and physical properties in RRMS and NMOSD patients vs healthy controls. (A, B)** Line graph and box plots display results of erythrocyte osmotic fragility and water solubility tests in RRMS and NMOSD patients compared to HC. Both RRMS and NMOSD patients showed significantly increased erythrocyte osmotic fragility, with more pronounced changes in RRMS patients (∗*P* < 0.05; ∗∗*P* < 0.01; ∗∗∗∗*P* < 0.0001; n=10 per group). **(C)** Heatmap reveals significant differences in plasma mitochondrial-related gene expression between RRMS patients and HC, as determined by qRT-PCR. n=4 per group. **(D)** ELISA demonstrates significantly elevated levels of oxidized DNA in plasma from RRMS and NMOSD patients compared to HC. Each group n=10 samples. **(E)** Immunoelectron microscopy shows increased 8-OHdG binding on the surface of deformed erythrocytes in RRMS patients, while NMOSD patients exhibit minimal 8-OHdG binding. Scale bar = 1.0 μm. **(F-H)** Erythrocyte marker Band3 co-localized with TLR9 and 8-OHdG more in RRMS and NMOSD patients than in healthy controls. Photographed at 40x. **(I)** Bar graph illustrates significantly increased binding of TLR9 to mitochondrial DNA on erythrocyte surfaces in RRMS patients by qPCR via the ChIP method.

Intriguingly, CD47—a critical "self-marking" molecule on erythrocytes—was significantly reduced in RRMS patients (*P* < 0.001) (Fig. 2d, f), suggesting a potential association between TLR9 upregulation, CD47 deficiency, and erythrocyte deformation. Such morphological aberrations may compromise erythrocyte function. Supporting this, osmotic fragility and water-soluble assays revealed pronounced pathological changes in erythrocyte fragility in RRMS patients (*P*<0.01) (Fig. 4a, b), whereas NMOSD patients exhibited only marginal alterations. These findings provide a plausible explanation for the erythrocytopenia and hemoglobin reduction uniquely observed in RRMS.

### Mitochondrial oxidative damage drives TLR9-ox-mtDNA interactions in RRMS erythrocytes

In aseptic inflammation, redistribution and dysfunction of TLR9 on the surface of erythrocytes are strongly correlated with serum levels of mtDNA. In MS, abnormal mitochondrial metabolism and increased release of ox-mtDNA are observed intracranially [13,35]. Our search in the GEO database revealed dysregulated mRNA expression in pathways related to mitochondrial autophagy, apoptosis, and catabolism in the peripheral blood of MS patients compared with healthy controls, as shown in the GSE136411 dataset (Fig. 1c, d). We analyzed 36 mRNA transcripts associated with mitochondrial metabolic functions for differential expression and revealed that 8 genes, such as *PINK1*, *HUWE1*, and *ATG7*, presented significant differences (*P*<0.05), whereas 24 genes presented moderate differences (*P*<0.1) in patients with MS (Fig. 1e), highlighting substantial mitochondrial metabolic dysfunction in MS.

To validate these findings, we conducted qRT-PCT analysis of the mRNA expression of 8 mitochondrial metabolism-related genes (e.g., *PINK1*, *HUWE1*, and *ATG7*) in plasma from RRMS patients and healthy controls. Consistent with those in the GSE136411 dataset, the expression levels of *PINK1*, *ATG7*, *RNF41*, and *VPS13D* were significantly elevated, whereas the *CDC37*, *BNIP3*, and *HUWE1* expression levels were notably reduced in RRMS patients (Fig. 4c). Given the impact of abnormal mitochondrial function on the release of ox-mtDNA [36,37], we employed 8-hydroxy-2'-deoxyguanosine (8-OHdG) as a marker of oxidized DNA [38,39]. ELISA confirmed significantly higher plasma levels of 8-OHdG in RRMS and NMOSD patients than in healthy controls (*P* < 0.0001) (Fig. 4d).

The potential relationship between elevated TLR9 expression on erythrocytes and ox-mtDNA levels remains unclear. Immunoelectron microscopy revealed significantly increased 8-OHdG levels in the altered erythrocyte membranes of both RRMS and NMOSD patients compared to healthy controls (Fig. 4e). IF staining further demonstrated enhanced co-localization of 8-OHdG and TLR9 on erythrocyte membranes (Band3) in RRMS and NMOSD groups (Fig. 4f-h). To investigate TLR9-ox-mtDNA interactions, we performed chromatin immunoprecipitation (ChIP) followed by qPCR, confirming strong binding between TLR9 and 8-OHdG (Fig. 4i). These findings provide compelling evidence that increased TLR9 expression on erythrocytes is closely associated with its binding to circulating ox-mtDNA in plasma.

### EAE Mice Exhibit Erythrocyte dysfunction and Hematopoietic Compensation linked to ox-mtDNA

**Figure 5:**
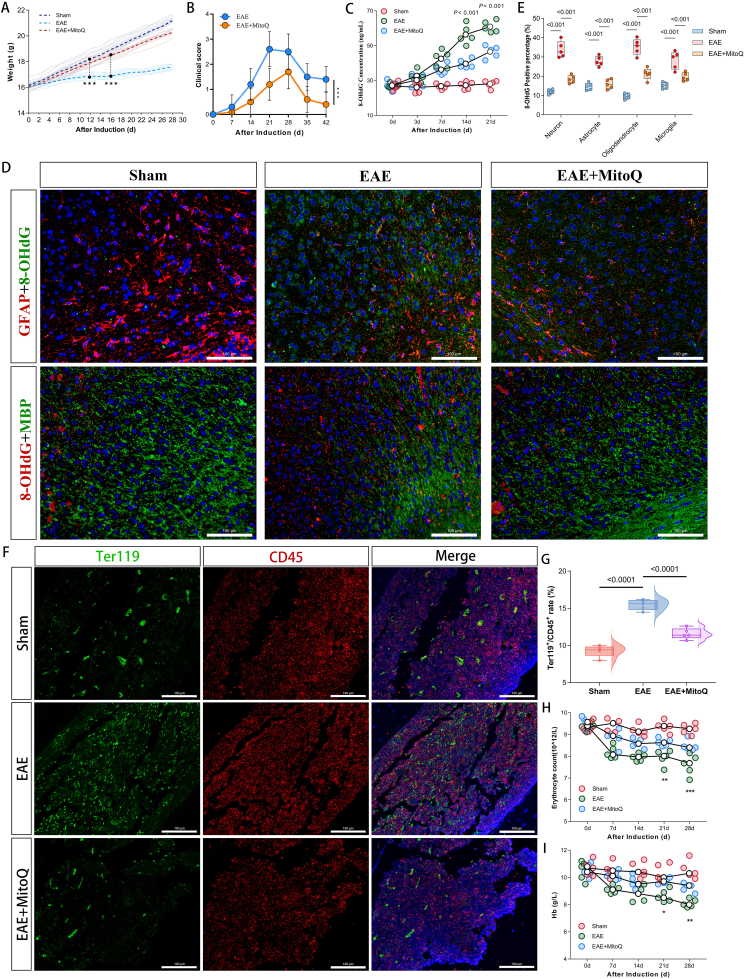
**Establishment of EAE mice model, cerebral mitochondrial oxidative damage and oxmtDNA release, and bone marrow erythroid progenitor cell proliferation. (A)** Line graph shows that EAE mice exhibited restricted weight gain starting from day 12 post-induction, while mitochondrial antioxidant MitoQ treatment significantly restored weight growth (n=6 mice per group; ∗∗∗*P*<0.001). **(B)** Line graph demonstrates that EAE mice reached peak clinical scores at day 21 post-induction, and MitoQ treatment ameliorated disease severity (n=10 mice per group). **(C)** ELISA reveals elevated plasma 8-OHdG levels in EAE mice compared to sham controls from day 3 post-induction, with progressive increase that was partially suppressed by MitoQ treatment. **(D)** IF staining shows increased colocalization of 8-OHdG (oxidative DNA damage) with astrocytes (GFAP) and oligodendrocytes (MBP) in the subcortical white matter of the mice striatum (a dense aggregation zone of oligodendrocytes and astrocytes) of EAE mice brains (40 × magnification). **(E)** Bar graph quantifies significantly elevated oxidative DNA release in neurons, astrocytes, oligodendrocytes and microglia of EAE mice versus sham controls at day 21 post-induction, which was inhibited by MitoQ treatment (5 regions analyzed). **(F,G)** At day 21 post-induction, EAE mice showed significantly increased erythroid progenitor cells (Ter119^+^/CD45^+^) in femoral bone marrow compared to sham controls, while MitoQ normalized this aberrant proliferation (40 × magnification; scale bar=100 μm). **(H,I)** Complete blood count analysis demonstrates persistent reductions in erythrocyte count and hemoglobin concentration during EAE progression, which were significantly improved by MitoQ treatment (n=5 per group; ∗*P*<0.05, ∗∗*P*<0.01, ∗∗∗*P*<0.001).

To further investigate the pathophysiological mechanisms underlying altered erythrocyte function in patients with RRMS, we established an EAE mice model to simulate the disease process of RRMS and evaluated the effects of mitochondrial antioxidant MitoQ treatment on erythrocyte dysfunction by mitigating mitochondrial damage. Continuous monitoring of body weight revealed that EAE mice exhibited significant growth retardation starting at 12 days post-induction (*P*<0.001) (Fig. 5a). Non-parametric testing confirmed that the clinical scores of EAE mice peaked at 21 days post-induction, with a median score of 2.5 (IQR 2.0–3.0). Most importantly, MitoQ treatment significantly reduced the clinical scores compared with the EAE mice (*P*<0.001) (Fig. 5b). In contrast, MitoQ-treated EAE mice showed improvements in both weight gain and neurological deficits. Histological analysis of striatal sections from EAE mice demonstrated scattered peripheral immune cell infiltration in the subcortical regions (sFig. 3a). IHC and LFB staining revealed extensive demyelination in the subcortical areas of EAE mice, which was ameliorated by MitoQ treatment (sFig. 3b, c), indicating that mitochondrial antioxidant therapy alleviated intracranial demyelination and inflammatory infiltration in EAE mice.

Mitochondrial antioxidant MitoQ links ox-mtDNA release to EAE inflammatory demyelination. We analyzed the level of oxidized DNA damage in neurons and various glial cells in the cerebral cortex and subcortical white matter—regions with high incidence of inflammatory demyelination in mice—via IF staining. The results showed that compared with sham controls, EAE mice exhibited significantly elevated 8-OHdG levels (a marker of oxidized DNA damage) in neurons (NeuN), astrocytes (GFAP), oligodendrocytes (MBP), and microglia (IBA-1) in both the cerebral cortex and subcortical regions (*P*<0.001). Among these, the increase in 8-OHdG levels was most pronounced in astrocytes and oligodendrocytes. MitoQ reversed these effects and ameliorated oxidative damage and demyelination (Fig. 5d, e; sFig. 2). Concurrently, ELISA analysis demonstrated that plasma 8-OHdG levels in EAE mice progressively increased from day 3 post-induction (*P*_3d_=0.02), and MitoQ treatment effectively reduced peripheral ox-mtDNA levels (Fig. 5c). These findings provide evidence that EAE mice develop progressive mitochondrial oxidative damage in the central nervous system, leading to sustained release of oxidized mitochondrial DNA into the peripheral circulation and subsequent elevation of plasma 8-OHdG levels.

Does elevated plasma ox-mtDNA affect erythrocyte parameters in mice? Compared to sham mice, EAE mice exhibited a significant reduction in RBC count and Hb levels starting from day 7 post-induction, though without overt anemia (*P*_RBC7d_<0.001, *P*_HB7d_<0.001) (Fig. 5h, i). These parameters were significantly improved in EAE + MitoQ mice (*P*_RBC7d_=0.006, *P*_HB7d_=0.02). However, no significant differences were observed in MCV, MCH, MCHC, or Hct among the EAE, EAE + MitoQ, and sham groups. These findings suggest a potential association between plasma ox-mtDNA levels and erythrocyte quantity during EAE pathogenesis.

Given that altered RBCs function and counts may influence bone marrow hematopoietic activity, we further examined erythropoiesis in the femoral bone marrow of EAE mice. Compared to sham mice, EAE mice displayed a slight increase in erythroid progenitor cells (CD45^-^;Ter119^+^) by day 21 post-induction (*P*<0.0001), indicating mild compensatory hematopoietic activation. This effect was not observed in EAE + MitoQ mice (Fig. 5f, g).

**Figure 6:**
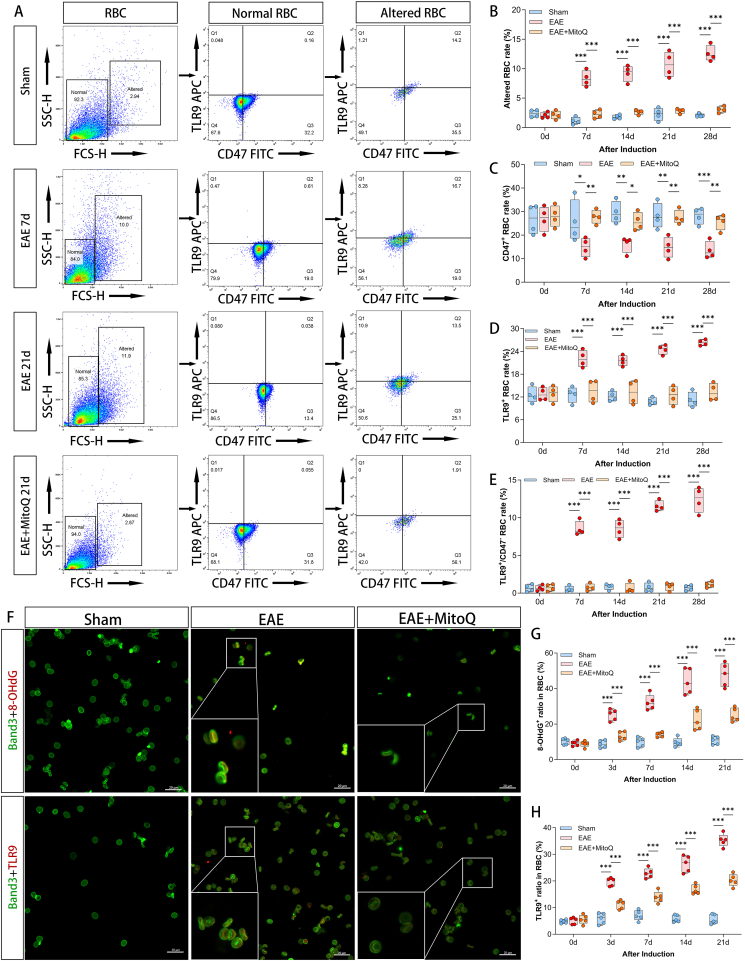
**Erythrocyte surface CD47 and TLR9 expression and their binding with 8-OHdG. (A-E)** Flow cytometry analysis revealed elevated TLR9 expression, reduced CD47 levels, and increased proportion of deformed erythrocytes in EAE mice, while MitoQ treatment ameliorated these pathological erythrocyte alterations. Each group of n=4 mice at each time point. **(F-H)** Immunofluorescence staining demonstrated progressively increased co-localization of TLR9 and 8-OHdG with Band3-labeled erythrocytes in EAE mice compared to sham mice from day 3 post-induction, with MitoQ treatment partially improving 8-OHdG binding and TLR9 expression. 5 different regions were taken for statistics. Scale bar=20 μm ∗*P*<0.05, ∗∗*P*<0.01, ∗∗∗*P*<0.001.

To verify whether EAE mice recapitulate the dynamic changes in erythrocyte surface proteins observed in RRMS patients, flow cytometry was performed. The results showed that, consistent with the erythrocyte characteristics of RRMS patients, EAE mice exhibited three key changes in erythrocytes: decreased CD47 expression, increased proportion of morphologically abnormal erythrocytes, and upregulated TLR9 positivity in abnormal RBCs (*P*_altered_<0.001, *P*_CD47_=0.0277, *P*_TLR9_<0.001). After MitoQ treatment, the number of abnormal erythrocytes in EAE mice decreased significantly, and the expression levels of CD47 and TLR9 returned to normal (Fig. 6a-e). IF staining further confirmed that, compared with mice in the Sham group, EAE mice showed a time-dependent increase in TLR9 expression and oxidized DNA levels on the erythrocyte surface starting from day 3 post-induction (*P*_TLR9_<0.0001, *P*_8-OHdG_=0.0002). MitoQ treatment significantly attenuated this increasing trend (Fig. 5f-h). Collectively, these results indicate that EAE mice develop erythrocyte pathological changes similar to those in RRMS patients. The origin of these changes is likely "intracranial inflammatory activation"—inflammatory activation triggers mitochondrial oxidative damage, promoting the release of ox-mtDNA into plasma. Elevated plasma ox-mtDNA levels, in turn, further lead to compensatory expansion of erythroid progenitor cells in the bone marrow and abnormal expression of erythrocyte surface proteins.

### Oxidized DNA-bound erythrocytes activate the splenic macrophage type 1 interferon pathway

Erythrocytes bound to ox-mtDNA exhibit insufficient CD47 expression and morphological changes. This characteristic may accelerate the phagocytosis of erythrocytes by splenic macrophages, while promoting the antigen-presenting function and inflammatory activation of macrophages. To evaluate the impact of this process on systemic inflammatory responses, we induced the differentiation of human monocytic leukemia cells (THP-1) into macrophages using phorbol 12-myristate 13-acetate (PMA) and co-cultured these macrophages with erythrocytes from RRMS patients or HC.

**Figure 7:**
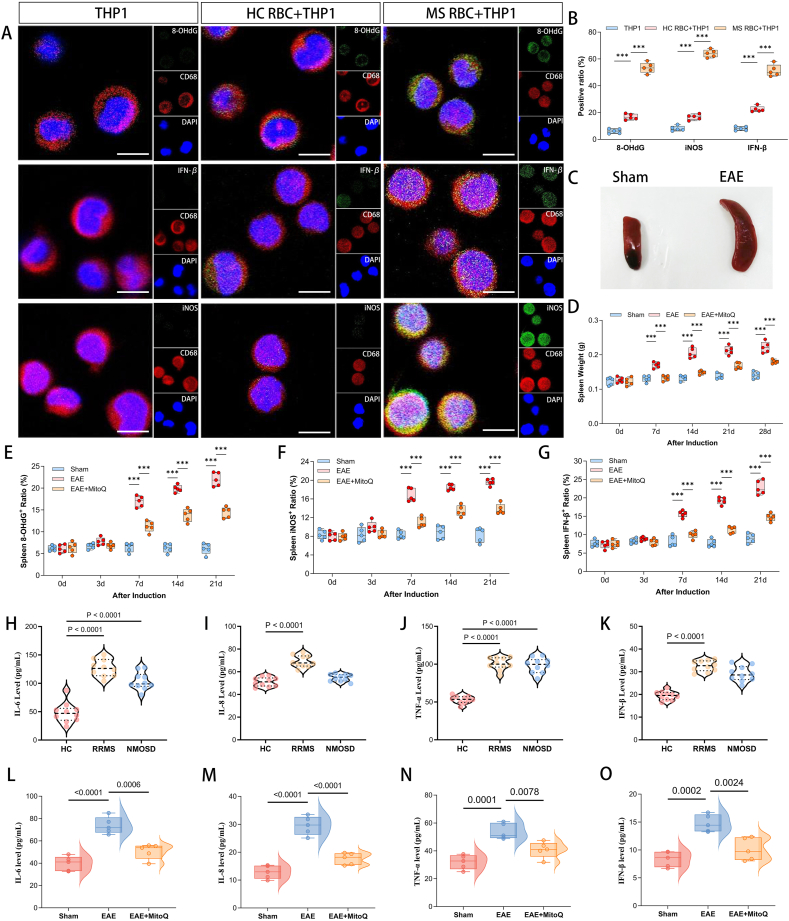
**Accelerated phagocytosis of erythrocytes and activation of the type 1 interferon pathway by splenic macrophages. (A, B)** IF staining revealed a marked reduction in erythrocyte numbers following co-culture of THP-1 cells with RRMS-derived RBCs, compared to THP-1-only or THP-1+HC-RBCs groups. THP-1 cells incubated with RRMS RBCs also exhibited significantly elevated levels of 8-OHdG-labeled oxidized DNA. Furthermore, these THP-1 cells showed increased iNOS expression and secreted higher levels of IFN-β. 5 independent regions were analyzed for quantification; scale bar = 10 μm. **(C, D)** EAE mice developed pathological splenomegaly compared to sham controls, which was significantly ameliorated by MitoQ treatment (∗*P*<0.05, ∗∗*P*<0.01, ∗∗∗*P*<0.001). **(E-G)** From day 7 post-induction, splenic macrophages from EAE mice showed increased 8-OHdG-labeled oxidized DNA and elevated iNOS expression, accompanied by enhanced IFN-β secretion—all of which were suppressed by MitoQ treatment. **(H-K)** RRMS and NMOSD patients displayed elevated plasma levels of type I interferon downstream proinflammatory cytokines (IL-6, IL-8, TNF-α, IFN-β) versus controls (n=10). **(L-O)** Similarly, EAE mice showed increased plasma levels of these cytokines, with MitoQ treatment inhibiting type I interferon pathway activation (n=5).

Detection after 3 h of co-culture showed that, compared with the THP-1 cells cultured alone group or the HC RBCs + THP-1 cells group, the RRMS RBCs + THP-1 cells group had a significantly higher erythrocyte clearance rate and a significantly increased 8-OHdG antibody positivity rate (*P*<0.001). In addition, the RRMS RBCs + THP-1 cells also exhibited obvious pro-inflammatory response, specifically manifested as increased intracellular interferon-β (IFN-β) expression (*P*<0.001) (Fig. 7a, b). This result suggests that macrophages can phagocytose ox-mtDNA-bound erythrocytes, thereby undergoing inflammatory activation and trigger type I interferon-mediated signaling pathways.

In EAE mice model, we further explored the impact of erythrocyte-ox-mtDNA interaction on splenic physiology functions. The result showed that EAE mice began to developed obvious splenomegaly from day 7 post-induction(*P*<0.001) (Fig. 7c, d), Which previously confirmed to be associated with macrophage-mediated erythrophagocytosis and inflammatory activation . Longitudinal observation of splenic pathology changes found that the 8-OHdG level in splenic macrophages of EAE mice increased significantly starting from day 7 (*P*_7d_=0.0005) (Fig. 7e, sFig4). Notably, this rise occurred after ox-mtDNA was observed binding to erythrocyte membranes. IF staining confirmed that splenic macrophages in EAE mice exhibited obvious pro-inflammatory activation and type I interferon pathway activation, specifically manifested as increased IFN-β expression. MitoQ treatment not only significantly reduced the ox-mtDNA level in the spleen but also alleviated the inflammatory activation of macrophages and the activation of the interferon pathway (*P*_7d_<0.001) (Fig. 7f, g, sFig4). This result indicates that during the progression of EAE, splenic macrophages are involved in the clearance of abnormal erythrocytes and the regulation of inflammatory responses, and MitoQ exerts a protective effect on both processes.

As expected from activated type I interferon signaling, plasma levels of IFN-β, interleukin-6 (IL-6), IL-8, and tumor necrosis factor-α (TNF-α) were elevated in EAE mice (*P*<0.001), which collectively confirmed a state of systemic inflammatory activation. MitoQ treatment restored these cytokine levels to normal (Fig. 7l–o). Similarly, the plasma of RRMS patients exhibited a parallel pro-inflammatory profile. Levels of IFN-β, IL-6, IL-8, and TNF-α were elevated compared to NMOSD patients, with the increases being more pronounced in RRMS (*P*<0.0001) (Fig. 7h–k). Our in vitro and in vivo validation confirms two key functions: macrophages phagocytose deformed erythrocytes, and erythrocytes themselves act as vehicles for ox-mtDNA. Together, these mechanisms mediate the activation of type I interferon-driven inflammation.

## Discussion

Our study provides a systematic characterization of erythrocyte abnormalities in MS and clarifies their underlying pathophysiological mechanisms. We achieved this through an integrated approach, combining clinical cohort analysis, molecular profiling, and validation in the EAE model. We establish that erythrocytes, unlike the traditional focus on lymphocytes, are active mediators in MS. Our data reveal that disease severity is linked to altered erythrocyte function, a consequence of mitochondrial damage causing TLR9-ox-mtDNA binding on erythrocytes. The subsequent clearance of these damaged cells by macrophages initiates a type I interferon-driven inflammatory response. This cycle, from erythrocyte injury to systemic inflammation, was interrupted by mitochondrial-targeted antioxidant treatment.

Erythrocytes have been known to possess immunoadhesive properties since the early 20th century [40]. The microenvironmental theory, erythrocytes may participate in local immune activation or maintenance of immune quiescence [41]. The role of erythrocytes in CNS autoimmunity remained unexplored [42]. Previous MS research has focused primarily on lymphocytes, leaving the role of erythrocytes in MS unclear. Clinical studies have reported conflicting findings on altered erythrocyte counts and properties [[43], [44], [45]].

Our study reveals that erythrocytes in MS undergo specific molecular reprogramming characterized by TLR9 membrane translocation and CD47 downregulation, leading to enhanced phagocytic clearance and the promotion of inflammatory responses. Clinically, the changes in erythrocyte parameters in MS patients are correlated with the severity of the disease, suggesting their utility as monitoring biomarkers. Data from the UK Biobank further indicate accelerated erythrocyte clearance in MS. While clinical evidence alone cannot establish causation, we have used EAE models and THP-1 assays to validate these findings experimentally. This can confirm that both the number and function of erythrocytes are altered during disease. Notably, these abnormalities were absent in peripheral demyelinating diseases such as MG and CIDP, suggesting that dysfunction of the erythroid TLR9-ox-mtDNA axis is a specific feature of inflammatory demyelinating disorders like MS. While recent studies have reported erythrocyte dysfunction in Alzheimer’s disease (AD), including alterations in membrane lipid composition, membrane fluidity, and β-amyloid deposition [46,47]. The specific TLR9-ox-mtDNA axis identified in MS has not been described in AD. These results highlight new directions for biomarkers and treatments, particularly mitochondrial-targeted therapies like MitoQ.

Glucocorticoids are known to increase erythrocyte count and hemoglobin levels via stimulating erythropoiesis and reducing erythrocyte clearance [48,49]. However, in our study, RRMS patients still exhibited significantly reduced erythrocyte parameters (RBC count, Hb, Hct) compared to healthy controls, despite blood collection after steroid effects had resolved. This finding further strengthens the conclusion that erythrocyte abnormalities in RRMS are an intrinsic feature of the disease rather than a secondary effect of steroid treatment.

Most studies of TLR function have focused on the role of TLRs in conventional immune cells (NK cells, B cells, etc.) [50,51]. However, some enucleated cells (e.g. platelets and erythrocytes) also express TLR9 on their surface [[52], [53], [54]]. Previous studies have shown that TLR9 expression on the surface of erythrocytes is associated with innate immune activation and erythrocyte clearance under inflammatory conditions [11,55]. Interestingly, we observed that the phenomenon of TLR9 translocation from the erythrocyte cytoplasm to the cell surface represents a potential adaptive response to oxidative stress injury. This phenomenon, together with the concomitant downregulation of CD47, collectively enhanced erythrocyte phagocytosis. Our analysis of splenic macrophages confirms this mechanism, showing that ox-mtDNA-bound erythrocytes not only accelerate clearance, but also actively drive interferon responses in splenic macrophages - a double-edged sword that may lead to both compensatory proliferation of erythroid progenitor cells and systemic inflammation.

Previous studies have demonstrated that inflammatory processes in the CNS during MS progression are associated with mitochondrial dysfunction and oxidative stress [56,57]. Following demyelination, mitochondria translocate from neuronal cell bodies to demyelinated axons, where their morphology becomes altered, initially increasing metabolic demand [58]. The disruption of the blood-brain barrier (BBB) constitutes an early pathological event in MS,facilitating the infiltration of immune cells and the subsequent leakage of inflammatory factors. Prior research has demonstrated that heightened BBB permeability during the acute phase of MS is intimately linked to the activation of glial cells, notably the secretion of matrix metalloproteinase-9 (MMP-9) by microglia. Although the release of ox-mtDNA may originate from oxidative damage in any tissue, during MS, the CNS undergoes oxidative injury due to activated inflammatory responses. Based on the results of IF staining, it is reasonable to hypothesize that the elevated levels of ox-mtDNA in plasma originate from oxidative damage within the CNS. Subsequently, this ox-mtDNA can cross the disrupted BBB and enter the systemic circulatory system. During the progression of MS, the peripheral blood concentrations of molecules associated with CNS damage—such as neurofilament light chain, glial fibrillary acidic protein (GFAP), and S100 Calcium-Binding Protein Beta (S100β)—may also increase. This phenomenon is directly attributed to the leakage of cerebrospinal fluid into peripheral tissue spaces following BBB impairment [59,60]. This observation further supports the hypothesis of *trans*-barrier migration of ox-mtDNA [61].

Existing studies have confirmed the presence of multiple mitochondrial abnormalities in MS, including increased mtDNA mutations, impaired mitochondrial gene expression, defective enzymatic activity, and reduced mtDNA repair capacity [[62], [63], [64]]. Our study found that the level of ox-mtDNA in the peripheral blood from RRMS patients was significantly elevated, establishing a direct correlation between plasma ox-mtDNA and mitochondrial oxidative damage in CNS neurons and glial cells. Notably, therapeutic intervention with the mitochondrial-targeted antioxidant MitoQ effectively suppressed the release of ox-mtDNA. MitoQ could reduce ox-mtDNA binding to erythrocytes, and significantly decreased the proportion of structurally abnormal RBCs. More importantly, this treatment exerted a protective effect on both the erythrocyte-macrophage antigen presentation cascade and subsequent inflammatory activation processes. As a mitochondrial-targeted antioxidant, the therapeutic effect of MitoQ in the EAE model is closely associated with its direct role in mitigating CNS mitochondrial oxidative damage, reducing ox-mtDNA release and inhibiting the erythrocyte-TLR9-ox-mtDNA axis. Meanwhile, it may also be related to the ability of MitoQ to prevent the initial activation of peripheral immune cells (e.g., macrophages, T cells) by reducing systemic oxidative stress and the initiation of inflammatory signals at the immunization stage.

The progressive loss of erythrocytes in RRMS patients might be driven by intracranial mitochondrial impairment. This condition triggers a cascade where ox-mtDNA release increases TLR9 and suppresses CD47 on erythrocyte surfaces. Previous studies have shown that TLR9 on erythrocyte membranes can activate macrophage inflammatory responses by binding unmethylated CpG-rich DNA. However, its role following ox-mtDNA binding remains poorly understood [10]. Our study offers preliminary evidence that erythrocytes carrying ox-mtDNA can trigger an inflammatory response in splenic macrophages. This activation involves the type I interferon pathway within the macrophages, which may subsequently drive systemic inflammation. Macrophages are known to drive tissue injury and chronic inflammation, which are central to MS pathogenesis. Therefore, inflammatory cascades mediated by erythrocytes could represent a further mechanism influencing disease progression. These findings expand the current understanding of erythrocyte-mediated immune regulation by elucidating the mechanistic role of erythrocytes in neuroinflammation through ox-mtDNA presentation.

Limitations: First, although UK Biobank data provided a large-scale observational basis, the core mechanistic validation was conducted using only 110 RRMS patients. This sample size may limit the generalizability of the findings across all MS subtypes. Future studies should include progressive forms (e.g., PPMS) to determine whether similar mechanisms operate. Second, although the patient and control groups were matched for age and sex subgroup analyses based on disease-modifying therapies (DMTs) were not performed. DMTs may affect erythrocyte parameters and inflammatory profiles. Third, since erythrocytes are anucleate and non-replicative, direct functional validation of TLR9 inhibition on erythrocytes was not feasible. Finally, the precise mechanisms governing the release of ox-mtDNA from the CNS into the periphery remain unclear and warrant further investigation. Future studies could explore whether inhibiting TLR9 trafficking via erythrocyte-derived vesicles modulates inflammatory activation. This would help clarify the role of erythrocytes in MS-related systemic immunity. Despite these limitations, the findings of this study underscore the potential significance of erythrocytes in MS pathophysiology and open new avenues for therapeutic targeting. Further research is essential to fully elucidate the contributions of erythrocytes to MS development. In future work, we aim to validate the erythrocyte pathogenic mechanisms, which were identified in the EAE model, in MS patients and to explore potential resultant therapeutic strategies derived from these mechanisms.

Our integrated clinical and experimental study establishes erythrocytes as active mediators in MS pathogenesis. We found that these cells are capable of binding and presenting ox-mtDNA that originates in the central nervous system. We demonstrate that ox-mtDNA, released following mitochondrial oxidative damage in neural cells, binds to erythrocyte TLR9. This binding induces CD47 downregulation and morphological alterations in the erythrocytes, promoting their clearance by the spleen. Through the erythrocyte-TLR9-ox-mtDNA axis, two pathways are activated: compensatory erythropoiesis is triggered, and macrophage type I interferon responses are initiated. Together, these effects fuel systemic inflammation. Notably, therapeutic intervention with MitoQ, a mitochondrial-targeted antioxidant, effectively disrupted this pathogenic cascade. It reduced the release of ox-mtDNA and helped preserve erythrocyte integrity, which ultimately led to suppressed neuroinflammation. Our findings suggest erythrocytes could link CNS damage to systemic inflammation, but this model remains to be proven. We propose that causal involvement of this cell type must be tested through specific interventions, such as erythrocyte-selective TLR9 inhibition. These findings enhance to our understanding of MS pathophysiology by highlighting a the potential role for erythrocytes in neuroimmune communication, and propose mitochondrial oxidative stress and erythrocyte-mediated inflammation as candidate targets for future therapeutic strategies. Further investigation in human clinical trials is warranted to assess the translational potential of erythrocyte parameters as biomarkers and mitochondrial protection as a treatment approach.

## Data availability

The datasets used and analyzed in this study are available from the corresponding authors upon request. Clinical data for all subjects are available as an additional file. This data can be found here: Researchers may have access to the UK Biobank dataset by submitting an application to the UK Biobank official website (https://www.ukbiobank.ac.uk/). This study used the UK Biobank resource with the application ID: 71051.

## Author contributions

YJJ, YFL, and ZG proposed the idea of this study and provided financial support. LFY and QMH participated in the design and execution of the experiments, analyzed the experimental data, and wrote the paper. YFC, HZQ, and YBY collected the clinical data and blood specimens. YDX, HML was responsible for the data collection and statistical analyses. QSL and ZYC participated in the execution of the animal experiments. All authors read and approved the final draft.

## Ethics Approval

Peripheral blood specimens were obtained from the First and Second Affiliated Hospital of Zhengzhou University. All patients provided written informed consent prior to sample donation. This study was reviewed and approved by the Ethics Committee of the First Affiliated Hospital of Zhengzhou University (Approval No. 2023-KY-1303-003; Date: June 5, 2024) and the Ethics Committee of the Second Affiliated Hospital of Zhengzhou University (Approval No. KY2025162; Date: April 14, 2025).

## Declaration of AI Assistance

No AI writing models or generative AI tools were used in the initial drafting of this manuscript, data analysis, result interpretation, or figure preparation. The DeepSeek model was employed exclusively for language polishing and academic English enhancement during the final revision stage. The authors assume full responsibility for the integrity and content of this work.

## Funding

This work was funded by the Science and Technology Basic Resource Survey Project of the 10.13039/501100002855Ministry of Science and Technology (China) of China (2018FY100900 to Yanjie Jia), the National Natural Science Foundation of China (82001290 to Zhe Gong), the 10.13039/501100006407Natural Science Foundation of Henan Province (232300420037 to Yanjie Jia),and the Joint Construction Project of the Medical Science and Technology Tackling Program of Henan Province (LHGJ20220332 to Yanfei Li).

## Declaration of competing interests

The authors declare that they have no known competing financial interests or personal relationships that could have appeared to influence the work reported in this paper.

[6].

## Abbreviations

MS: multiple sclerosis
RRMS: relapsing-remitting multiple sclerosis
NMOSD: neuromyelitis optica spectrum disorder
MG: myasthenia gravis
AE: autoimmune encephalitis
CIDP: chronic inflammatory demyelinating polyradiculoneuropathy
MOGAD: Myelin oligodendrocyte glycoprotein antibody-associated disease
CNS: central nervous system
TLR9: Toll-like receptor 9
INF: interferon
EDSS: Expanded Disability Status Scale
ox-mtDNA: Oxidized mitochondrial DNA
EAE: Experimental autoimmune encephalomyelitis
8-OHdG: 8-hydroxy-2'-deoxyguanosine
THP-1: Human monocytic leukemia cells
