# Supplementary material for: Altered Erythrocyte Function via TLR9-ox-mtDNA Binding Links Mitochondrial Oxidative Damage to Systemic Inflammation in Multiple Sclerosis
Source: Neurotherapeutics. 2026 Mar 12;23(2):e00863. doi: 10.1016/j.neurot.2026.e00863 (PMC12996651; doi:10.1016/j.neurot.2026.e00863)
Supplement: Multimedia component 1 [file mmc1.docx]

**Supplementary Material**

**Supplementary Method**

**Luxol fast blue (LFB) staining**

LFB staining of EAE mice brain sections was performed following a standard protocol. In brief, the sections were immersed in LFB staining solution at 60℃. Subsequently, differentiation occurred in 95% ethanol followed by 70% ethanol, and the samples were rinsed in distilled water to halt differentiation. The sections were then counterstained in eosin solution, dehydrated, fixed, and sealed before observation.

**Osmotic fragility assay and hemolysis assays.**

Osmotic fragility and hemolysis assays were performed following established methods. Freshly isolated RBCs (5×10^5^) from MS patients, NMOSD patients, and healthy donors were washed in 1 ml of PBS, resuspended in 100 µl of PBS, and incubated at room temperature in 15 ml of NaCl solutions (0.9%, 0.8%, 0.7%, 0.6%, 0.3%, 0.2%, 0.1%, or 0% NaCl) in deionized water for 15 min. The mixture was then centrifuged at 800×g for 5 min at 4℃. The hemoglobin content in the supernatant was determined via a QuantiChrome hemoglobin assay. Osmotic fragility was calculated relative to the hemoglobin content of RBCs incubated in 0% NaCl, which was set to 100%, and the data were normalized accordingly.

**Flow cytometry**

Peripheral blood samples from both MS subjects and EAE mice were processed within 2 h of collection for initial isolation and purification. A total of 2.5 × 10⁶ purified erythrocytes were resuspended in PBS and incubated for 1 hour with a mixture of FITC-conjugated CD47 (2 µg, clone CC2C6, BioLegend) and APC-conjugated TLR9 (0.25 µg, S16013D, BioLegend) antibodies to facilitate binding to their respective target antigens on the erythrocyte membrane. Appropriate controls, including unstained cells and samples stained separately with either FITC-CD47 or APC-TLR9 antibodies, were used to establish optimal laser settings on the flow cytometer (NOVOCYTE 3130, ACEA Biosciences) and to compensate for potential spectral overlap between the FITC and APC channels. Data analysis was performed using FlowJo software version 10.6.2. Representative flow cytometry plots of unstained and single-stained controls (FITC and APC channels) for subject and EAE mice RBCs are shown in sFig5.

**Immunohistochemical chemistry**

Immunohistochemical staining was used to analyze myelin basic protein (MBP) expression in EAE mice brain tissues. First, the brain sections were subjected to antigen retrieval via citrate buffer and then incubated in PBS containing 0.1% Triton X-100 and 3% BSA for 1 h. Next, the sections were incubated overnight at 4 ℃ with the primary antibody against MBP (1:500; Cell Signaling Technology, 78896s). The corresponding secondary antibody was subsequently applied at room temperature for 1 h. Finally, the sections were dehydrated, fixed, and sealed after they were restained with hematoxylin and differentiated in hydrochloric acid alcohol.

**Immunofluorescence staining**

Immunofluorescence staining of 2.5 × 10^6^ purified erythrocytes from subjects and mice was performed via standard procedures. The brain, spleen, and femur bone marrow tissue sections were dried and stained for immunofluorescence. The primary antibodies used included those against Band3 (1:200, Santa Cruz, sc-133190), TLR9 (1:100, Santa Cruz, sc-47723), 8-OHdG (1:200, Bioss, bs-1278r), NeuN (1:300, Cell Signaling, 94403s), IBa-1 (1:200, Abcam, ab-283319), MBP (1:200, Cell Signaling, 78896s), GFAP (1:200, Proteintech, 60190-1-Ig), TLR9 (1:100, Santa Cruz, sc-47723), IFN-β (1:200, Proteintech, 27506-1-AP), CD68 (1:100, Santa Cruz, sc-47723), Ter-119 (1:200, Thermo Fisher, MA1-70078), iNOS (1:200, Proteintech, 22226-1-AP), and CD45 (1:100, Bioss, bs-4819r). The samples were incubated with secondary antibodies against the corresponding species for 1 h at room temperature. The cell nuclei were restained with DAPI (Servicebio, G1012). A laser confocal microscope (Zeiss LSM880 Airyscan) was used to capture images of the brain, spleen and femur bone marrow sections and RBCs.

**Chromatin immunoprecipitation (ChIP)**

The RBCs were fixed with 1% formaldehyde for 10 min, and the mixture was stopped by adding glycine to a final concentration of 0.125 M. The RBCs were washed with cold PBS and lysed in RIPA-LS buffer for 15 min. Sonication was performed via a Biosensor Pico for 20 cycles of 30 s each with a 30-s pause. The lysate was incubated with MagStrep "type 3" XT beads or control protein A beads for 3 h. The beads were washed twice with RA-LS buffer, twice with RIPA-HS buffer, and once with RIPA-LI buffer. The precipitated TLR9 was washed with 1x biotin elution buffer. The sample was decrosslinked overnight at 65 ℃ by adding 500 mM NaCl to the eluent and protease K. For qPCR validation, mtLOOP, mtCOX2, and mtND1 probes were used.

**Enzyme-linked immunosorbent assay (ELISA)**

According to the manufacturer's instructions, an ELISA kit (Elabscience, Wuhan, China) for 8-OHdG, IL-8, IFN-β, IL-6, and TNF-α was used to quantify the plasma levels of these markers from both the subjects and the EAE mice. The plasma samples were added to 96-well plates coated with specific antibodies, followed by incubation with peroxidase and biotin to form immune complexes. The absorbance measurements were read at 450 nm via a SpectraMax M5e ELISA reader (Molecular Devices, San Jose, USA) to determine the standard concentrations.

**Supplementary Figures and Tables**

**sTable1.**General information and clinical indicators of other patients.

|  | **MG(n=24)** | **Encephalitis(n=21)** | **CIDP(n=7)** | **MOGAD(n=8)** |
| --- | --- | --- | --- | --- |
| **General** Information | | | | |
| Sex(M:F) | 15:9 | 12:9 | 4:3 | 3:5 |
| Age,years,mean(±SD) | 52.16±15.60 | 43.95±15.31 | 55.85±21.31 | 34.50±10.12 |
| **Laboratory** test results | | | | |
| RBC,mean±SD,10^12/L | 4.49±0.47 | 4.10±0.59 | 4.26±0.36 | 4.48±0.36 |
| WBC,median(IQR),10^9/L | 6.98(5.45-8.96) | 6.84(5.42-8.43) | 10.76(10.03-11.27) | 9.82(7.04-13.47) |
| PLT,median(IQR),10^9/L | 230.00(190.00-263.00) | 237.00(205.00-259.50) | 223.00(210.00-341.00) | 257.00(160.25-320.75) |
| Hb,mean±SD,g/L | 135.06±13.11 | 125.64±18.53 | 136.20±11.10 | 134.75±11.02 |
| RDW,median(IQR),% | 13.70(12.90-33.72) | 13.50(12.95-15.07) | 12.98(12.30-40.00) | 12.80(12.30-13.50) |
| HCT,mean±SD,LL | 0.40±0.03 | 0.37±0.05 | 0.40±0.02 | 0.41±0.02 |
| MCV,mean±SD,fL | 89.76±6.83 | 91.82±585 | 94.50±3.29 | 90.97±4.70 |
| MCH,median(IQR),pg | 30.90(29.67-32.30) | 30.60(29.46-32.30) | 3200(30.10-32.90) | 30.50(28.27-31.40) |
| MCHC,median(IQR),g/L | 338.00(330.50-345.75) | 336.00(331.05-338.90) | 333.10(330.00-350.00) | 330.00(326.25-335.00) |
| N%,mean±SD,% | 63.77±11.99 | 64.05±15.14 | 75.51±16.11 | 75.15±14.87 |
| L%,mean±SD,% | 27.43±11.42 | 27.77±13.17 | 18.45±12.45 | 18.76±12.11 |
| TB,mean±SD,μmol/L | 9.48±3.75 | 8.16±3.19 | 9.72±2.66 | 8.93±4.63 |
| CB,mean±SD,μmol/L | 4.03±1.49 | 3.89±1.26 | 4.07±0.53 | 3.67±161 |
| ALT,median(IQR),U/L | 18.00(15.00-32.75) | 18.00(12.00-30.50) | 42.00(16.00-49.00) | 26.00(10.25-46.00) |
| AST,median(IQR),U/L | 20.00(14.25-27.50) | 18.00(13.50-20.00) | 22.00(13.00-33.00) | 15.50(13.00-20.00) |
| GGT,median(IQR),U/L | 22.50(14.25-30.00) | 32.00(16.50-60.50) | 33.00(19.00-45.00) | 25.50(22.00-51.50) |
| TP,mean±SD,g/L | 66.33±7.44 | 65.60±5.49 | 70.35±10.15 | 67.42±6.26 |
| ALB,median(IQR)g/L | 41.75(39.10-43.37) | 38.90(37.70-43.75) | 42.60(37.60-4780) | 42.55(39.22-45.52) |
| Cr,median(IQR),μmol/L | 69.00(53.00-80.00) | 57.0048.50-65.50) | 57.00(50.00-60.00) | 62.5048.00-78.25) |
| BUN,median(IQR),mmol/L | 4.90(4.04-5.84) | 3.703.00-4.38) | 4.80(4.50-6.70) | 4.70(3.42-5.75) |
| UA,mean±SD,μmol/L | 272.49±110.68 | 249.33±102.29 | 273.00±100.78 | 246.62±101.50 |
| PT,median(IQR),s | 11.35(11.00-11.77) | 11.80(10.60-12.45) | 10.90(10.40-12.50) | 11.65(11.10-12.40) |
| NR,median(IQR) | 1.03(0.99-1.06) | 1.07(0.97-1.12) | 0.99(0.95-1.13) | 1.05(1.01-1.15) |
| APTT,median(IQR),s | 28.10(27.02-28.77) | 27.30(25.70-3025) | 25.30(24.50-31.70) | 27.50(25.77-30.12) |
| TT,median(IQR),s | 15.30(13.95-16.75) | 15.30(13.55-16.75) | 14.60(13.90-15.90) | 15.10(13.57-17.30) |
| D-Dimer,median(IQR),mg/L | 0.19(0.07-0.37) | 0.17(008-0.36) | 0.10(0.06-0.37) | 0.09(0.06-0.19) |

The table lists the basic information and clinical test results of 24 patients with myasthenia gravis, 21 patients with autoimmune encephalitis, 7 patients with CIDP, and 8 patients with MOGAD.

**sTable2** Differences in clinical data between patients in the <5 years and ≥5 years disease duration groups.

|  | **＜5years（n=88）** | **≥5years（n=22）** | ***P*** |
| --- | --- | --- | --- |
| Sex(M:F) | 64:24 | 18:4 | 0.381 |
| Age,years | 41(29.5-52) | 38.5(34.75-49) | 0.908 |
| months of oneset | 12(1.25-25) | 96(72-120) | ＜0.001*** |
| EDSS | 2(1-3) | 2.5(1.87-3.62) | 0.12 |
| RBC,10**^**12/L | 4.33±0.43 | 4.09±0.35 | ＜0.05* |
| Hb,g/L | 129.3±15.9 | 118.2±16.3 | ＜0.05* |
| Hct,L/L | 0.390±0.042 | 0.363±0.041 | ＜0.05* |
| RDW,% | 12.85(12.5-13.5) | 13.25(12.37-15.06) | 0.116 |
| MCV,fL | 90.8(87.82-93.5) | 92.05(85.72-93.77) | 0.905 |
| MCH,pg | 30.3(29.02-31.5) | 30.1(28.25-31.22) | 0.241 |
| MCHC,g/L | 332(327-339.75) | 325(319.25-335.5) | ＜0.05* |

**sTable3** Differences in clinical data between patients with mild and severe neurological impairment.

|  | **Mild（n=84）** | **Severe（n=26）** | ***P*** |
| --- | --- | --- | --- |
| Sex(M:F) | 58:26 | 24:2 | ＜0.05* |
| Age,years | 41(28.25-49.75) | 38.5(32.75-53.25) | 0.59 |
| months of oneset | 24(2-48) | 44(11.25-63) | 0.098 |
| EDSS | 1.5(1-2) | 4(3.87-4.12) | ＜0.001*** |
| RBC,10**^**12/L | 4.39(4.13-4.66) | 3.87(3.52-3.94) | ＜0.001*** |
| Hb,g/L | 128.8(121-141.7) | 117(107-123.2) | ＜0.001*** |
| Hct,L/L | 0.392(0.366-0.421) | 0.355(0.331-0.364) | ＜0.001*** |
| RDW,% | 13.1(12.5-13.9) | 12.8(12.4-13.8) | 0.451 |
| MCV,fL | 90.14(87.15-93.07) | 92.67(90.02-96.05) | ＜0.05* |
| MCH,pg | 30.1(29-31.17) | 31.1(29.77-31.77) | ＜0.05* |
| MCHC,g/L | 331(324-338) | 331.5(323-338.8) | 0.88 |

**sTable4** Primer sequences for mitochondrial metabolism-related genes in qPCR and mitochondrial genes in CHIP.

| **Primer Information** | **Primer name** | **Primer sequence（5'-3')** |
| --- | --- | --- |
| NM_032409.3 | H-PINK1-S | CCTCGTTATGAAGAACTATCCCTG |
|  | H-PINK1-A | GGATGTTGTCGGATTTCAGGTC |
| NM_007065.4 | H-CDC37-S | AGTGCCAGAGGAAACTGAAGG |
|  | H-CDC37-A | ACCATGCTCTTGCTGAAGCC |
| NM_004052.3 | H-BNIP3-S | AGGGCTCCTGGGTAGAACTG |
|  | H-BNIP3-A | TCTCTCCAATGCTATGGGTATCTG |
| NM_031407.7 | H-HUWE1-S | GGTGCTGGCAGTCCTCAATCT |
|  | H-HUWE1-A | TGCCAAATGTTGTAGCCGAGTT |
| NM_006327.4 | H-TIMM23-S | GGGGCACTTTGGGCTAATAC |
|  | H-TIMM23-A | ACCACCTCGTGCTATCCCT |
| NM_015378.4 | H-VPS13D-S | ATTGAGGAGAGTAAACGATGGACC |
|  | H-VPS13D-A | TGGAGTTATCTTGGGTGTTCGTC |
| NM_001242826.2 | H-RNF41-S | GTCCGGCTTGACAACCTCAT |
|  | H-RNF41-A | GACGTCTTCTCCAGCTCTGC |
| NM_001136031.3 | H-ATG7-S | TAGTAGTGCCTTGGATGTTGGG |
|  | H-ATG7-A | CAGCCCAGCAGAGTCACCATT |
| NM_002046 | H-GAPDH-S | GGAAGCTTGTCATCAATGGAAATC |
|  | H-GAPDH-A | TGATGACCCTTTTGGCTCCC |
| NC_012920.1 | H-D-LOOP-S | GGATACCAACAAACCTACCCACC |
|  | H-D-LOOP-A | GATGTCGGATACAGTTCACTTTAGC |
| NC_012920.1 | H-MT-CO2-S | ACTACGGCGGACTAATCTTCAACT |
|  | H-MT-CO2-A | AGCATTGACCGTAGTATACCCCC |
| NC_012920.1 | H-MT-ND1-S | TCCTAATGCTTACCGAACGAAA |
|  | H-MT-ND1-A | ATGGTAGATGTGGCGGGTTT |

**
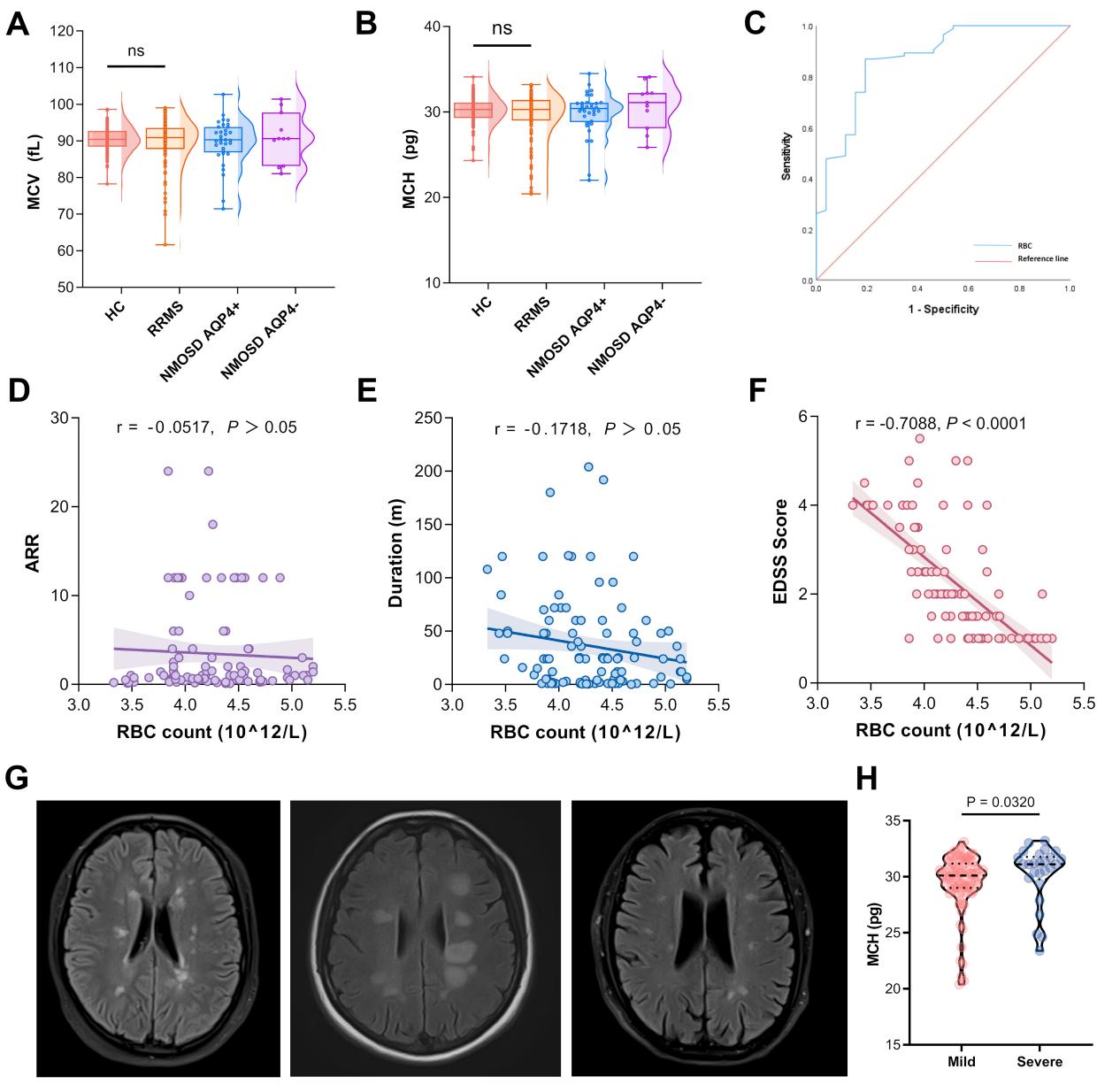
**

**sFig 1.** Analysis of erythrocyte variability and correlation

**(A, B)** The comparisons of MCV and MCH between the four groups are presented separately. There were no significant differences in MCV and MCH between control and RRMS patients. **(C)** The area under the ROC curve (AUC) for RBC count in identifying severe neurological impairment in RRMS was 0.870 (95% CI: 0.788–0.952, *P*<0.001), with an optimal cutoff value of 3.965, yielding a sensitivity of 0.869 and a specificity of 0.808. **(D-F)** Spearman correlation analysis was performed to assess the relationship between RBC count and EDSS score, annualized relapse rate, and disease duration in RRMS patients. The results showed that RBC count was significantly correlated with EDSS score (r=-0.7088, *P*<0.0001), but no significant correlation was observed with disease duration or ARR. **(G)** MRI of three patients with RRMS. **(H)** Subgroup analysis based on EDSS scores showed that RRMS patients with severe neurological impairment had higher MCH levels.


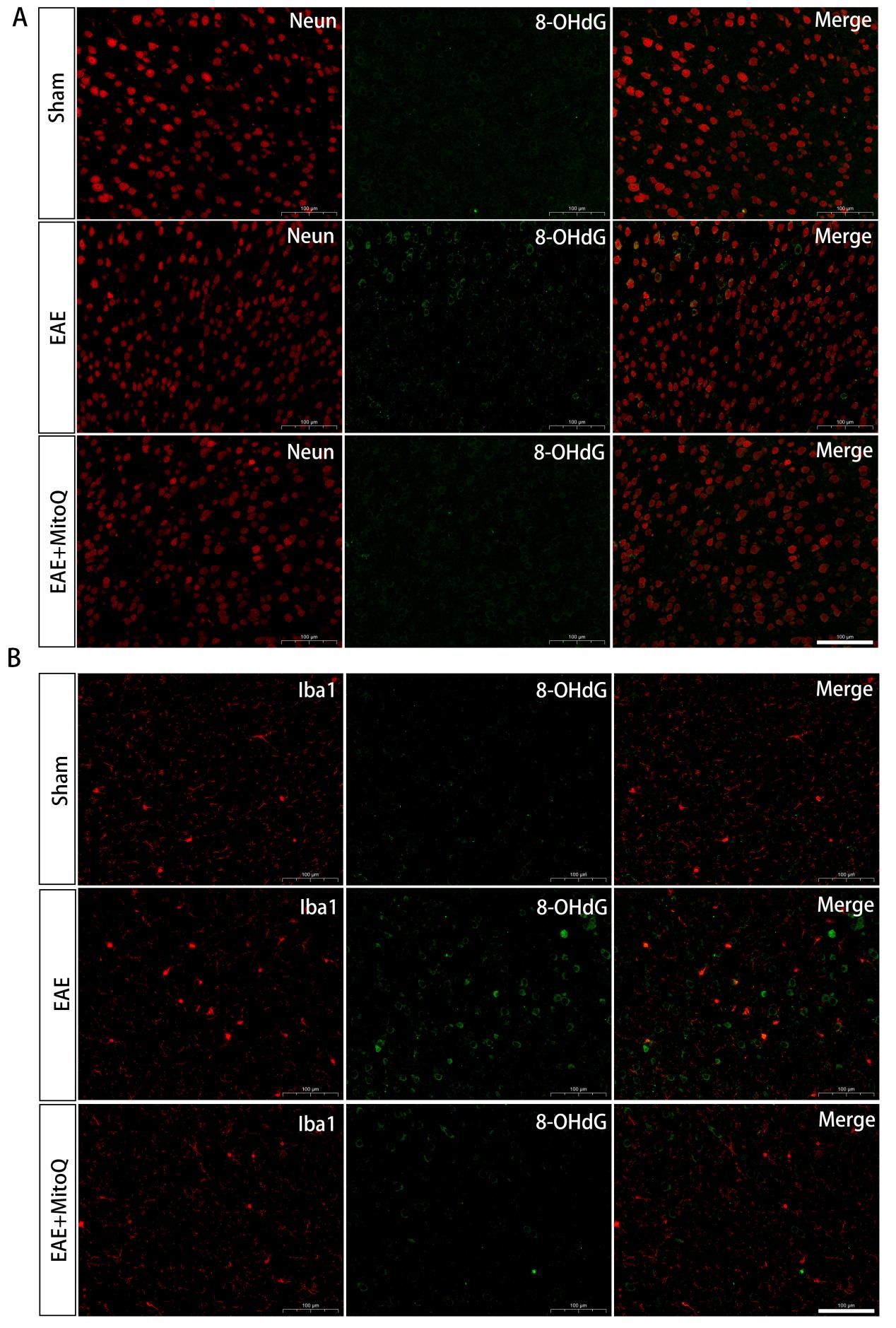


**sFig 2.** Oxidized DNA expression in neurons and microglia at the striatal level in sham, EAE and EAE+MitoQ mice brains. 40× magnification; scale bar=100 μm.


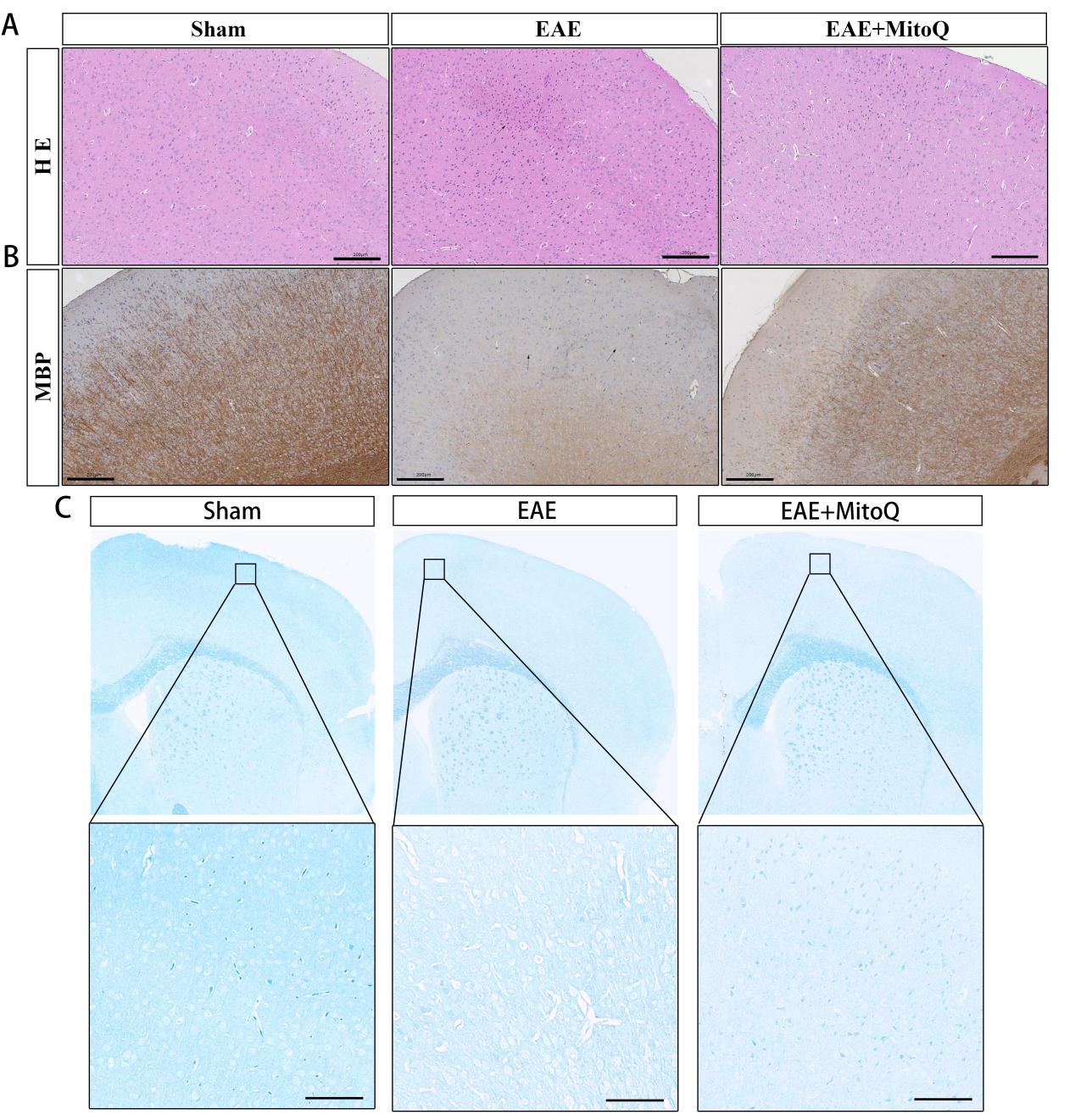


**sFig 3. Inflammatory infiltration and demyelination changes in brain tissues of EAE mice. (A)** HE staining showed peripheral immune cell infiltration in the subcortex at the striatal level of the brain in EAE mouse. Scale bar=200 μm. **(B)** IHC staining revealed MBP-labeled myelin deficiency in the subcortex of the brain of EAE mouse. Scale bar=200 μm. **(C)** LFB staining showed myelin deficiency and vacuole formation in the subcortex of the brain of EAE mouse. Scale bar=100 μm. After treatment with MitoQ, inflammatory cell infiltration in the subcortical region was reduced, and demyelination showed significant improvement.


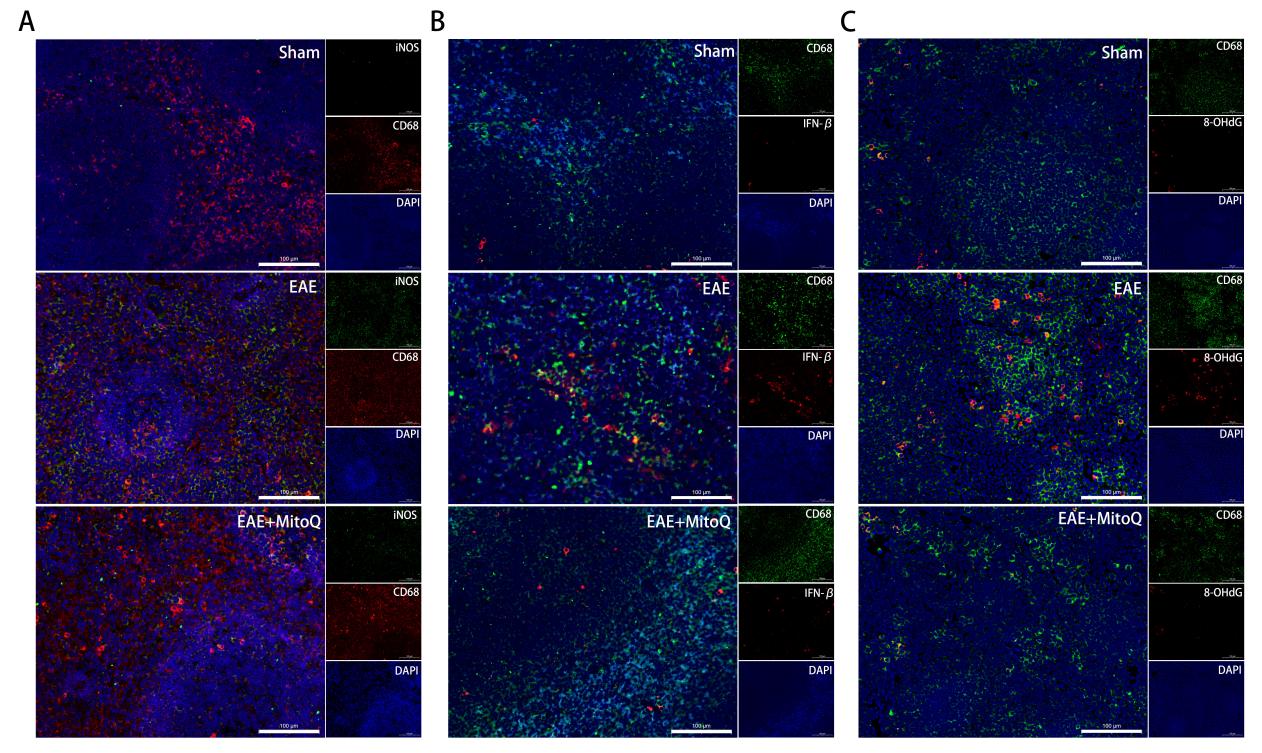


**sFig 4.** After 7 d of induction, EAE mouse had elevated levels of 8-OHdG-labeled oxidized DNA in splenic macrophages and more iNOS-labeled M1-type-polarized macrophages, which secreted more IFN-β, compared with sham mouse. MitoQ treatment reduced oxidative DNA and suppressed type I interferon-mediated inflammatory activation. Scale bar=100 μm.


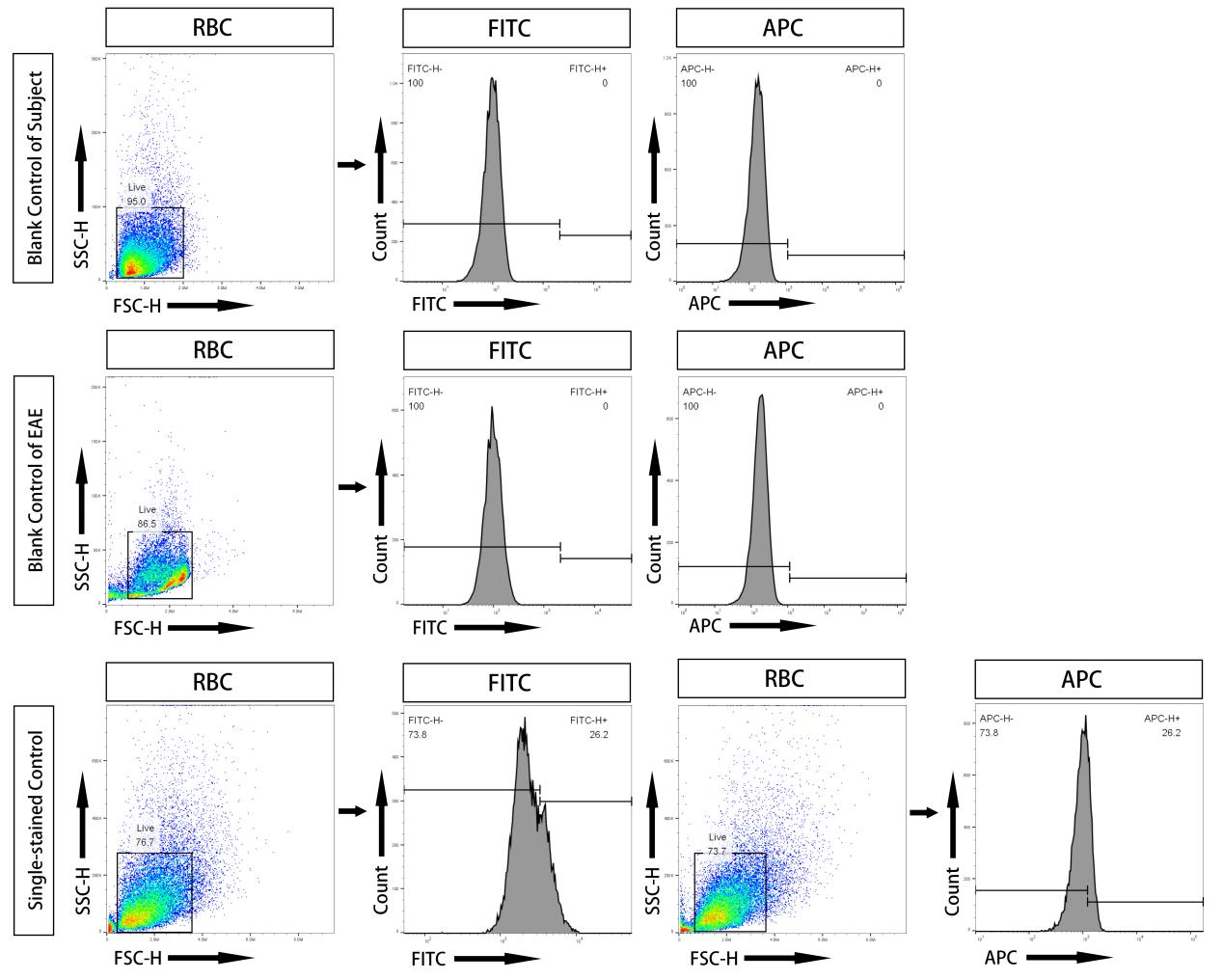
**sFig 5.** Compensation settings were established using unstained and single-stained controls (FITC and APC) from both subjects and EAE mice erythrocytes. Subsequently, these settings were applied to the analysis of erythrocytes co-stained with FITC-CD47 and APC-TLR9 antibodies.
